# Impact of CRISPR/Cas9-Mediated CD73 Knockout in Pancreatic Cancer

**DOI:** 10.3390/cancers15194842

**Published:** 2023-10-03

**Authors:** Jinping Zhang, Shuman Zhang, Isabella Dörflein, Xiaofan Ren, Susanne Pfeffer, Nathalie Britzen-Laurent, Robert Grützmann, Xianglong Duan, Christian Pilarsky

**Affiliations:** 1 Department of Surgery, Universitätsklinikum Erlangen, Friedrich-Alexander Universität Erlangen-Nürnberg (FAU), 91054 Erlangen, Germany; jinpingzhang.med@outlook.com (J.Z.); zhang_shu_man@163.com (S.Z.); isabella.doerflein@uk-erlangen.de (I.D.); renxiaofan1013@gmail.com (X.R.); susanne.pfeffer@uk-erlangen.de (S.P.); nathalie.britzen-laurent@uk-erlangen.de (N.B.-L.); robert.gruetzmann@uk-erlangen.de (R.G.); 2 Second Department of General Surgery, Shaanxi Provincial People’s Hospital, Xi’an 710068, China; duanxianglong@nwpu.edu.cn; 3 Second Department of General Surgery, Third Affiliated Hospital of Xi’an Jiaotong University, Xi’an 710068, China; 4 Institute of Medical Research, Northwestern Polytechnical University, Xi’an 710072, China

**Keywords:** pancreatic cancer, CRISPR/Cas9, CD73, deep sequencing

## Abstract

**Simple Summary:**

Pancreatic cancer is one of the cancers with the highest mortality rate, and targeted therapy against specific genes that play a crucial role in cancer is the latest therapeutic approach. This study showed that CD73 is highly expressed in pancreatic cancer, and the application of CRISPR/Cas9 technology to knockout of CD73 in human and murine cell lines can inhibit the proliferation and migration of tumor cells and induced G1 cell cycle arrest. We also found that CD73 deletion inhibited the ERK/STAT3 pathway and activated the E-cadherin pathway. Pbk, Fastk, Cdk19, Adck5, Trim28, and Pfkp are the genes that may regulate CD73 in pancreatic cancer.

**Abstract:**

Pancreatic cancer is among the cancers with the highest mortality rates. Most of the patients are found to have advanced cancer, losing the chance of surgical treatment, and there is an urgent need to find new treatment methods. Targeted therapy for specific genes that play a key role in cancer is now an important means to improve the survival rate of patients. We determined that CD73 is highly expressed in pancreatic cancer by flow cytometry and qRT-PCR assays combined with bioinformatics techniques. Application of CRISPR/Cas9 technology to knockout CD73 in human and murine cell lines, respectively, revealed that CD73 inactivation inhibited cell growth and migration and induced G1 cell cycle arrest. We also found that CD73 deletion inhibited the ERK/STAT3 pathway and activated the E-cadherin pathway. In addition, a CRISPR/Cas9 protein kinase library screen was performed and identified Pbk, Fastk, Cdk19, Adck5, Trim28, and Pfkp as possible genes regulating CD73.

## 1. Introduction

Pancreatic cancer is currently the seventh leading cause of cancer deaths globally. In Europe, it is surpassed only by lung, colorectal, and breast cancers in terms of mortality among all cancers [1]. Cancer registry data from 185 countries predicted that there would be 466,003 deaths from cases of pancreatic cancer globally in 2020 [2]. In addition, pancreatic cancer mortality is projected to continue to rise in the coming decades, with more than 800,000 deaths expected by 2040 [3,4]. Surgery, chemotherapy, radiotherapy, and targeted therapy are currently the most popular treatments for pancreatic cancer, with surgical resection being the only completely curative treatment [5]. However, there are no symptoms in the early stage of pancreatic cancer. When apparent signs and symptoms appear, it often indicates that the disease has reached an advanced stage, making surgical treatment impossible [6]. Therefore, the search for a new treatment for pancreatic cancer is of great importance.

In recent years, tumor immunotherapy has gained widespread attention, and its combination with other traditional therapies can significantly improve the survival rate of patients [7]. Tumor immunotherapy mainly includes tumor vaccines, cytokine therapy, adoptive immunotherapy, and immune checkpoint blockade, among which, the immune checkpoint blockade method appears to be most efficacious [8]. The immune system can identify tumor cells and clear the tumor through a series of immune responses [9]. Immune checkpoints are regulators of the immune system that prevent the immune system from indiscriminately attacking cells. However, some cancers can protect themselves against attack by stimulating immune checkpoint targets [10,11]. Therefore, the identification of immune checkpoint proteins is the key to immunotherapy. The roles of CD73, CD80, CD154, CD252, and CD276 as common immune checkpoints have been widely validated in cancer [12,13,14,15,16]. However, there are relatively few studies about them in pancreatic cancer.

CD73 has been one of cancer research’s most critical immune checkpoints in recent years. CD73, also known as ecto-5′-nucleotidase or NT5E, is an enzyme expressed on the surfaces of various cells. It plays a vital role in maintaining the homeostasis of the immune system, and its production of free adenosine inhibits the cellular immune response, thus promoting the immune escape of tumor cells [17]. It is also involved in the regulation of T cells [18]. CD73 is overexpressed in many cancer tissues, including those of breast, colorectal, and gastric cancer, and high CD73 expression in tumors often predicts a poor prognosis [19]. Many studies have validated CD73 as a critical molecule in cancer development [20,21,22]. CD73′s function and mechanism of action in pancreatic cancer are not yet clear.

In this study, we examined the expression of the aforementioned immune checkpoint genes in pancreatic cancer and confirmed that, among them, CD73 is highly expressed in pancreatic cancer. We used CRISPR/Cas9 technology to knockout CD73 in pancreatic cancer in vitro, aiming to explore the function and mechanism of action of CD73. In addition, we performed a protein kinase CRISPR/Cas9 screen in TB32047 cells to identify genes that may affect CD73 expression.

## 2. Materials and Methods

### 2.1. Cell Culture

This study used human and murine pancreatic cancer cells (PANC1 and TB32047). PANC1 (CRL-1469™) PDAC cells were purchased from the ATCC^®^ (American Type Culture Collection). SUIT2 cells (JCRB1094, RRID: CVCL_3172) were purchased from the Japan Research Bioresource Cell Bank. Miapaca2 (RM-CRL-1420TM, RRID: CVCL_0428), SU86.86 (CRL-1837TM, RRID: CVCL_3881), Aspc1 (CRL-1682TM, RRID: CVCL_0152) cell lines were purchased from ATCC. David Chang and Debabrata Mukhopadhyay kindly donated TKCC-10 and Mayo4636 cell lines. David Tuveson of CSHL (Cold Spring Harbor Laboratory, Cold Spring Harbor, NY, USA) kindly provided the primary mouse pancreatic cancer cell lines TB32047, a murine cell line derived from a KPC model. The KPC792 cell line was obtained from Marc P. Stemmler. All cell lines were cultured at 37 °C in a humidified environment with 5% CO_2_. Cells from the HEK293TN cell line were also obtained from the ATCC^®^. All cells were washed with Dulbecco’s Phosphate-Buffered Saline (DPBS) without Cacl_2_ and Mgcl_2_ (Cat# 14190094, Gibco™, Darmstadt, Germany) and 0.25% trypsin-ethylenediaminetetraacetic acid (EDTA) (Cat# 25200-072, Gibco™) for collection. Cells were frozen in basal medium with 20% fetal bovine serum (FBS) (Cat# A3160802, Gibco™) and 10% dimethyl sulfoxide (DMSO) (Cat# D2650-5X5ML, Sigma-Aldrich Chemie, Darmstadt, Germany). We generated the TB32047-Cas9 and PANC1-Cas9 stable cell line by virally transducing lentiCas9-blast (Addgene, Cambridge, MA, USA; Cat# 52962) into the cells and selecting them using 10 μg/mL of blasticidin (InvivoGen, Cat# ant-bl-1, Toulouse, France) for 3 days. We verified Cas9 expression by Western blot analysis.

### 2.2. Flow Cytometry

The cells were washed twice with DPBS, digested with trypsin, and harvested. The cells were resuspended with FACS buffer to a concentration of 1 × 10^7^ cells/mL (1 million/100 µL). Transfer of the resuspended cells (1 million/100 μL) into a 5 mL FACS tube was then performed, and fluorescently conjugated antibody (1:100) (see Table S1) was added. We then gently moved a pipette up and down to help disperse the doublet (15–20 times) and incubated it for 20–30 min (at 4 °C) in the dark. The cells were washed with 500 μL FACS buffer and centrifuged for 5 min (350× *g*) at 4 °C. The supernatant was then discarded, and the cells were lysed in 200 μL FACS buffer in a 5 mL FACS tube. We then analyzed the stained cell samples using flow cytometry (BD Biosciences LSRII, Heidelberg, Germany), and we analyzed the flow cytometry data using FlowJo™ v10.8 software (BD Life Sciences).

### 2.3. FACS Sorting

Cells were collected and stained with CD73 antibody, as described in Section 2.2, Flow Cytometry. Cells were transported, on ice, to the FACS facility and sorted with FACS Aria II. After FACS sorting, cells with high levels of CD73 expression were collected, and they continued to be cultured for a new round of sorting. Cells were again collected after three rounds of sorting. To identify the sgRNAs integrated into the cells with high levels of CD73 expression, 10 million cells were then collected for genomic DNA isolation.

### 2.4. Lentivirus Production

We seeded 7 × 10^6^ HEK293TN packaging cells in T75 flasks and incubated the cells at 37 °C, 5% CO_2_, for 24 h. Transfection was performed with Lipofectamine™ 3000 Transfection Reagent (Cat. # L3000001, Invitrogen, Darmstadt, Germany). We prepared a mixture of the transfection plasmids (2.8 µg pMDLg/pRRE, 1.4 µg pRSV-REV, 1.4 µg pMD2.G, and 4.3 µg library plasmid) with P3000 and Opti-MEM. (See Table S2 for a list of the plasmids.) The pooled sgRNA library (A modification of mouse Brie kinome pooled library additionally containing sgRNA against pancreatic cancer target genes (ACF-library), See Table S7) targeting the murine kinome was a gift from John Doench and David Root [23] (RRID: Addgene_75316, Addgene, Cambridge, MA, USA), and it was modified for the inclusion of pancreatic-cancer-related genes by our lab. The library contained 3446 sgRNAs for 915 murine genes. Then, we gently added the mixture to the Opti-MEM with Lipofectamine™3000. The mixture was incubated for 15–20 min at room temperature, and the transfection mix was carefully transferred to T75 flasks. The cells were then incubated for 6 h, and the media was carefully aspirated. The media was then replaced with 12 mL DMEM complete medium. The virus was harvested 24 h post-transfection, and the viral supernatant was centrifuged at 2000× *g* for 10 min to remove the cell pellets. As soon as was possible, the viral supernatant was stored at −80 °C after it was filtered through a 0.45 μm PES filter to avoid a loss of titer.

### 2.5. Lentivirus Transduction

A quantity of 1.8 × 10^6^ TB32047 Cas9 cells was seeded into T75 flasks with 12 mL medium on the day before transduction. After 24 h, the medium was removed from the cells and replaced with 0.1% polybrene medium with ACF-library lentivirus. The regular medium was introduced to the flask after the lentivirus had been present for 24 h. The cells were then incubated for another 2 days, and the medium was replaced with 10 µg/mL puromycin for 72 h. Finally, the cells were trypsinized and counted after puromycin selection. Based on the cell count results, we then calculated the required volume of the virus (MOI = 0.3–0.5).

### 2.6. Deep Sequencing and Data Analysis

Genomic DNA was isolated with a NucleoSpin Blood L Kit (Cat# 740954.20, MACHEREY-NAGEL, Düren, Germany), followed by a PCR procedure to amplify the sgRNAs. A quantity of 20 µg of DNA was amplified using P5 (5′-ACACTCTTTCCCTACACGACGCTCTTCCGATCTNNNNNTCTTGTGGAAAGGACGAAACACCG-3′) and P7 (5′-GTGACTGGAGTTCAGACGTGTGCTCTTCCGATCTTCTACTATTCTTTCCCCTGCACTGT-3′) primers. For each sample, Q5 Master Mix (Cat# M0494S, Biolabs, Beverly, MA, USA) was used to conduct two independent 100 µL reactions with 10 µg of genomic DNA in each reaction, and next-generation sequencing was performed on the Illumina HiSeq 2500 platform in the Deep Sequencing Facility of TU Dresden. The raw data of the deep sequence were analyzed using PinAPL-Py [24].

### 2.7. CRISPR/Cas9 Gene Editing

CD73 was knocked out in the TB32047 and PANC1 cell lines with the CRISPR/Cas9 gene-editing plasmid pSpCas9(BB)-2A-Puro (PX459) V2.0 (PX459) and sgRNA designed for CD73. pSpCas9(BB)-2A-Puro (PX459) V2.0 was a gift from Feng Zhang (Addgene plasmid # 62988; http://n2t.net/addgene:62988; accessed on 16 January 2023, RRID:Addgene_62988) Ligated vectors were transferred into Endura sterile cells (Cat# 60242-1, Lucigen, Middleton, WI, USA) for plasmid extraction. CRISPR/Cas9 plasmid target CD73 was transfected with Lipofectamine™3000 for 24 h, followed by selection with puromycin (10 µg/mL) for 3 days. After growth, Western blotting and qRT-PCR were performed to verify the knockout. Wild-type cells were designated as WT cells, while the negative control cells and the group of CD73-negative cells are referred to as NC cells and KO cells, respectively.

The primers designed for the sgRNAs in this study included:

mm-CD73-sg1-forward: 5′-CACCgCCCACTCAGACGTGCCGCTTC-3′, reverse: 5′- AAACGAAGCGGCACGTCTGAGTGGC-3′,

mm-CD73-sg2-forward: 5′-CACCgCCTCTAGCACATCAGATATC-3′, reverse: 5′- AAACGATATCTGATGTGCTAGAGGC-3′,

hu-CD73-sg1-forward: 5′-CACCgCGCCCTGCGCTACGATGCCA-3′, reverse: 5′- AAACTGGCATCGTAGCGCAGGGCGC-3′, and

hu-CD73-sg2-forward: 5′-CACCgTGTGGACGTCGTGGTGGG-3′, reverse: 5′- AAACCCCACCACGACGTCCACACC-3′.

### 2.8. Confirmation of CRISPR/Cas9-Mediated Knockout

NucleoSpin^®^ Tissue (MACHEREY-NAGEL, Düren, Germany) was used to isolate DNA from the cell lines. PCR products for sequencing were amplified using primers listed in Table S3. PCR fragments were cloned in pMiniT 2.0 with the NEB^®^ PCR Cloning Kit (New England Biolabs, Frankfurt, Germany, E1202). For each single-cell clone, 10 bacterial colonies were chosen, and plasmid DNA was isolated (GeneJET Plasmid Miniprep Kit, K0503, Thermo Fisher, Langenselbold, Germany) for sequencing in Eurofins Genomics.

### 2.9. Quantitative PCR

Total RNA was extracted from the cells using the NucleoSpin^®^ RNA Plus kit (MACHEREY-NAGEL, Cat# 740984.250). RNA (1000 ng) was reverse-transcribed using a High-Capacity cDNA Reverse-Transcription Kit (Applied Biosystems^TM^, Cat# 4368814, Darmstadt, Germany) according to the manufacturer’s protocol. β-Actin and GAPDH (mouse and human) expression levels were used to normalize RNA input levels. Power SYBR™ Green PCR Master Mix (Thermo Fisher Scientific, Waltham, MA, USA) was used for all genes of interest. (qRT-PCR primers are listed in Table S4). The mRNA expression levels of different genes were quantified using a CFX96 system. Gene expression levels were calculated using the 2^−ΔΔCT^ method. 

### 2.10. Western Blot Analysis

Cells were lysed in RIPA buffer (Cat# 89900, Thermo Fisher Scientific, Waltham, MA, USA) containing 1% protease and phosphatase inhibitors (Cat# 78442, Thermo Fisher Scientific). Protein concentrations were quantified using a BCA Protein Assay Kit (Cat# 23250, Thermo Fisher Scientific). A quantity of 20 µg of protein samples was loaded onto 4–12% NUPAGE Bis-Tris gels (Cat# NP0322BOX; Thermo Fisher Scientific) using a Mini Gel Tank chamber system (Invitrogen), and the proteins were transferred to Amersham™ Protran™ Premium 0.45 µm NC membranes (Cat# GE10600003, Cytiva, Freiburg, Germany). After blocking with 5% milk, primary antibodies (listed in Table S5) were used according to the manufacturer’s requirements and incubated overnight at 4 °C. HRP-conjugated anti-rabbit or anti-mouse IgG was used as a secondary antibody. Signal quantification was performed with an Amersham Imager 600 (Pittsburgh, PA, USA) and SignalFire™ ECL reagent (Cat# 6883S, Cell Signaling Technology Europe, Leiden, The Netherlands). We individually performed all Western blot assays 3 times. 

### 2.11. Cell Cycle Analysis and Apoptosis Assay

The cells were appropriately harvested and washed in cold PBS. Then, cold 70% ethanol was added while the cells were shaken. They were stored at −20 °C overnight for preservation. Then, they were washed 2 times with PBS and incubated with RNase (100 µg/mL) for 60 min at 37 °C. They were then stained with propidium iodide (PI; 50 µg/mL, Cat# 421,301, BioLegend, Amsterdam, The Netherlands). Quantitative analysis was performed using flow cytometry on a BD Biosciences LSRII flow cytometer.

Adherent cells and cells in the culture medium were collected for apoptosis detection and stained with an FITC-Annexin V Apoptosis Detection Kit I (Cat# 556547, BD Pharmingen, Heidelberg, Germany) according to the manufacturer’s instructions. FITC-Annexin V uptake was measured on a BD Biosciences LSRII flow cytometer. Flow cytometry results were analyzed using FlowJo™ v10.8 software (BD Life Sciences).

### 2.12. Proliferation Assay

A quantity of 1 × 10^3^ cells/well of TB32047 and PANC1 cells was plated in black 96-well plates. Cells were cultured for 6 days and stained with DAPI (Hoechst 33,342, Cat# H3570, Life Technologies, Darmstadt, Germany) every 24 h. Then, photographs of every well were taken using an EVOS FL Auto 2 imaging system (Cat# AMAFD2000, Invitrogen, Darmstadt, Germany). Cells in these images were counted using HCS Studio Cell Analysis software v2.0 (Thermo Fisher Scientific, Cat# SX000041A, Waltham, MA, USA). Each data point was produced in triplicate and normalized to the counts from Day 1, and each experiment was run three times (*n* = 3).

### 2.13. Colony Formation Assay

Each cell type was seeded in three wells of 6-well plates (100 cells/well). TB32047 cells were cultured for 7 days, and PANC1 cells were cultured for 14 days. The medium was removed, the cells were fixed with 4% formaldehyde solution for 10 min, and the formaldehyde was discarded. The cells were stained with 0.1% crystal violet for 15 min and washed with water. Then, the colonies were counted. Colonies consisting of more than 50 cells were counted visually, and the average number of colonies was calculated.

### 2.14. Wound Healing Assay

TB32047 cells, at 3.5 × 10^5^ per well, and PANC1 cells, at 4 × 10^5^ per well, were seeded in 12 wells for the wound-healing experiments. Then, they were cultured and allowed to grow for 24 h. After the cells reached 100% confluence, the serum-free medium was replaced, and the cells were starved for 6 h. A 100 µL tip was used to create a wound in each well of the plate. Then, we replaced the medium with 1% FBS medium (for TB32047) or 2% FBS medium (for PANC1). Each circular wound within the field of view was photographed with an EVOS microscope at 0 h and again at 24 h, and ImageJ software (https://imagej.nih.gov/ij/, accessed on 16 January 2023) was used to measure the notch area and to calculate the percentage of healing.

### 2.15. Immunofluorescence

10^5^ Cells were grown in 4-well chamber slides, and we observed the cell growth status to ensure their uniform distribution and density. Fixed with 4% formaldehyde for 15 min, permeabilized with 0.1% Triton (Sigma-Aldrich) for 30 min; non-specific antigens were blocked with 10% Goat Normal Serum (GNS). The above steps were carried out at room temperature. E-cadherin (CST, 3195, RRID: AB_2291471) antibody (1:50–1:100) diluted in 5% GNS was added, and then they were incubated overnight at 4 °C and Isotype Normal Rabbit IgG (RD, AB-105-C) was utilized as a control. The following day, the slides were washed 2 times with TBS 1X, for 5 min each time. We then added secondary anti-rabbit Alexa Fluor 488 (Invitrogen, A11034, RRID: AB_2576217, Eugene, OR, USA) diluted at 1:500 in 5% GNS and incubated them for 1 h in the dark at RT. DAPI (Thermo Scientific, V13069211, Eugene, OR, USA) diluted at 1:5000 (in water) was added, and the slides were incubated for 10 min. Fluorescent mounting medium (DAKO) and a coverslip were then added. Images were acquired and processed using an Evos FL Auto 2 imaging system (Invitrogen by Thermo Fisher Scientific, Bothell, WA, USA). Quantitative analysis of E-cadherin staining at cell–cell junctions was completed using ImageJ (https://imagej.nih.gov/ij/, accessed on 16 January 2023).

### 2.16. Statistical Analysis

Values are expressed as mean ± SEM or mean ± SD (GraphPad Prism 8.0). Unless indicated, results are from two or three independent experiments. Statistical significance was determined using two-way ANOVA or one-way ANOVA (or a mixed model). *p*-values are reported in the graphs, with * *p* < 0.05, ** *p* < 0.01, *** *p* < 0.001, and **** *p* < 0.0001. In all analyses, *p*-values < 0.05 were considered to be statistically significant.

## 3. Results

### 3.1. CD73 Is Highly Expressed in Pancreatic Cancer and Reduces Patient Survival

**Figure 1:**
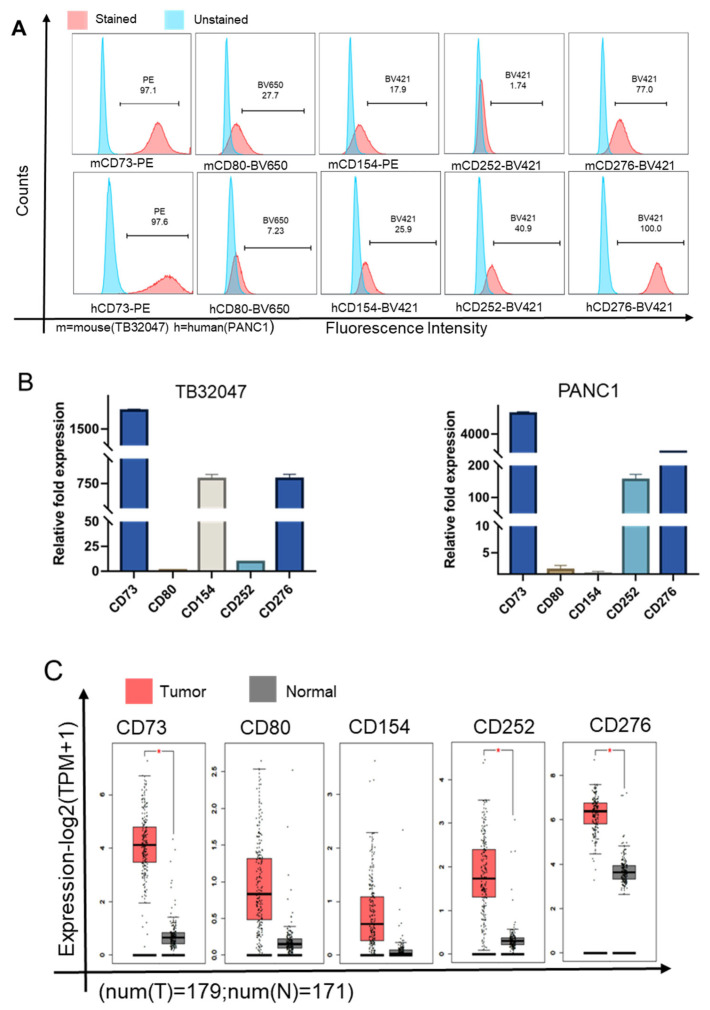

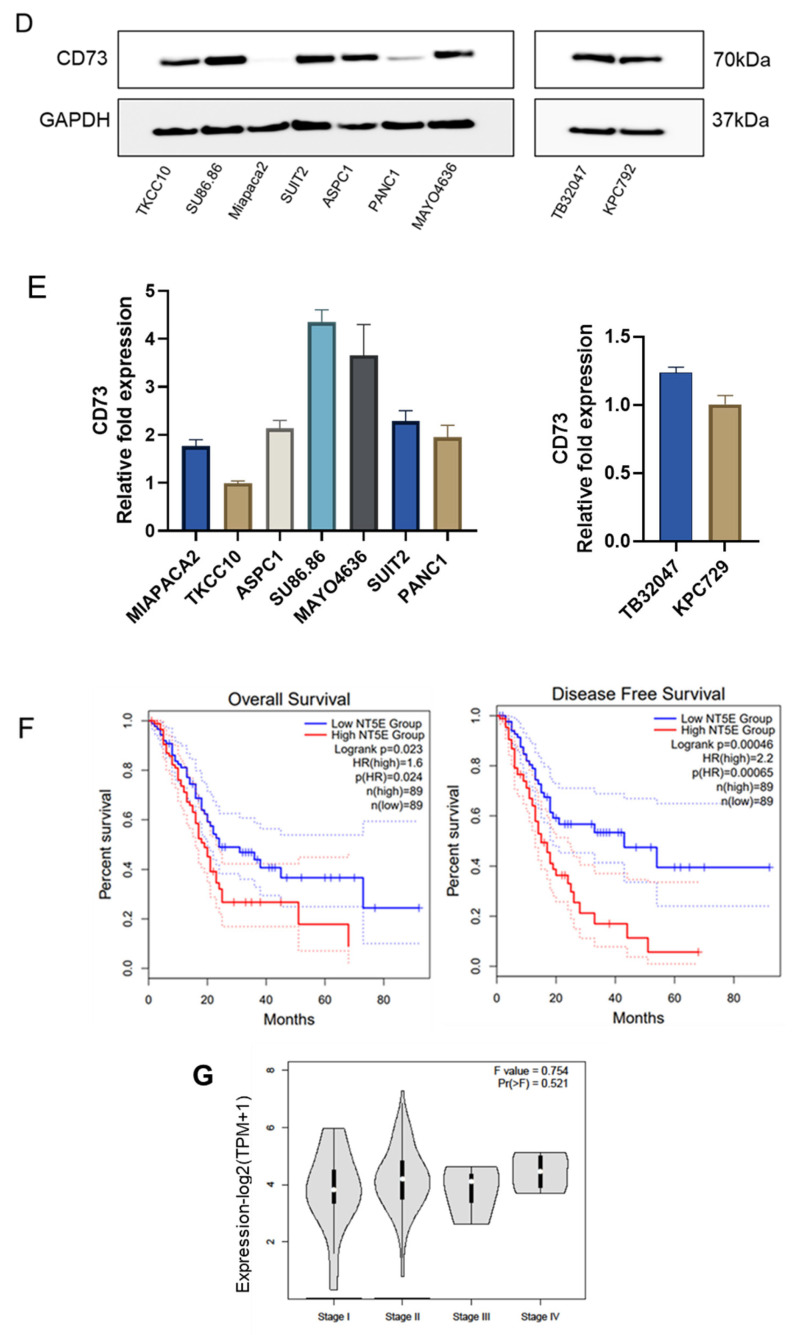
CD73 is highly expressed in pancreatic cancer: (**A**) Flow cytometry was performed to detect levels of CD73, CD80, CD154, CD252, and CD276 expression on the surface of the cell membrane of TB32047 cells and PANC1 cells; (**B**) Expression of the CD73, CD80, CD154, CD252, and CD276 mRNAs of TB32047 cells and PANC1 cells. CD154 was used as the control in the TB32047 cell. The PANC1 cell control was CD80; (**C**) Gene Expression Profiling Interactive Analysis (GEPIA) was performed to validate the CD molecules in PAAD samples, as compared with normal samples. The red boxes indicate the cancer tissue groups, the gray boxes indicate the normal tissue groups, and * *p* < 0.05; (**D**) Western blot analysis of CD73 expression levels in human and mouse PDAC cell lines; (**E**) qRT-PCR showed the expression of CD73 in different PDAC cell lines; TKCC10 was used as the control in the human cell lines. The mouse cell lines’ control was KPC729. (**F**) Data from GEPIA2 were applied to survival analysis. Kaplan–Meier survival analysis shows that higher CD73 expression is associated with poor overall survival and poor disease-free survival; dotted lines indicate the confidence interval (**G**) Analysis of CD73 expression levels in different pathological stages of pancreatic cancer using GEPIA2. The uncropped blots are shown in Supplementary File S1.

First, using flow cytometry, we analyzed the expression of CD molecules in TB32047 and PANC1 cells, namely CD73 (97.1%), CD80 (27.7%), CD154 (17.9%), CD252 (1.74%), and CD276 (77%), as compared with respective, unstained controls in TB32047 cells, and CD73 (97.6%), CD80 (7.23%), CD154 (25.9%), CD252 (40.9%), and CD276 (100%), as compared with respective, unstained controls in PANC1 cells (Figure 1A). The expression levels of surface CD73 are higher than those of the other CD molecules in TB32047 and PANC1 cells. qRT-PCR showed that the mRNA expression of CD73 is the highest in TB32047 cells and PANC1 cells (Figure 1B). CD73, CD252, and CD276 were upregulated in pancreatic cancer compared to normal tissues by GEPIA2 analysis (Figure 1C). Therefore, we focused on CD73, which is highly expressed in PDAC, for further analysis.

To further determine the expression of CD73 in pancreatic cancer, we examined CD73 by Western blot and qRT-PCR analysis in seven human cell lines (TKCC10, SU86.86, MIAPACA2, SUIT2, ASPC1, PANC1, and MAYO4363) and two mouse cell lines (TB32047 and KPC792). The results showed that in the human PDAC cell line, the expression of CD73 in SU86.86 cells was the highest, and in MIAPACA2 cells was the lowest; CD73 was also expressed in PANC1 cells and TKCC10 cells. In the mouse PDAC cell line, the expression of CD73 in TB32047 cells was higher than in KPC792 cells. qRT-PCR also showed that the mRNA level of CD73 is highest in the SU86.86 cells and was relatively in the middle in PANC1 cells. The qRT-PCR result of mouse cell lines is consistent with western blotting (Figure 1D,E). Data from GEPIA2 indicate that increased CD73 expression is statistically associated with poor overall survival and poor disease-free survival in PDAC (Figure 1F). However, we found no difference between the expression of CD73 in different pathological stages of pancreatic cancer (Figure 1G). Therefore, we selected the PANC1 cell line, which has relatively intermediate CD73 expression in human pancreatic cancer cell lines, and the mouse pancreatic cancer TB32047 cell line for further study.

### 3.2. Expression of CD73 after CRISPR/Cas9-Mediated Knockout in TB32047 Cells and PANC1 Cells

**Figure 2:**
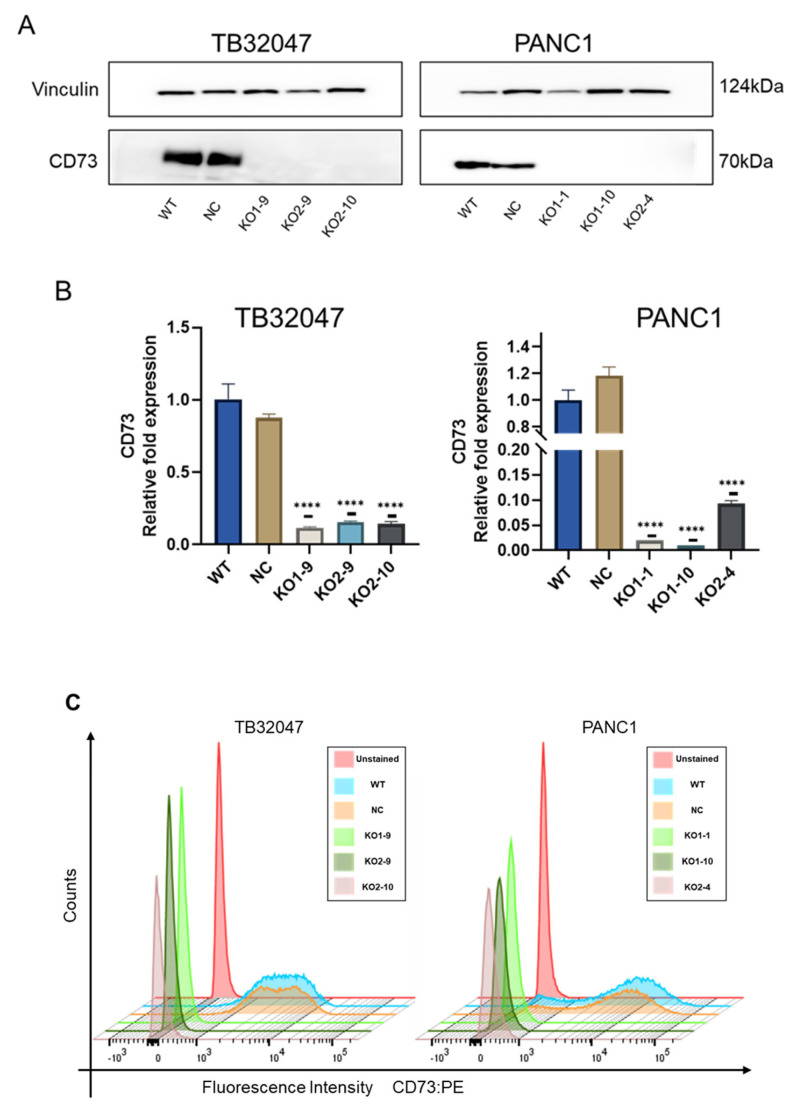
CD73 expression by wild-type cells, negative controls, and clones in TB32047 and PANC1 cell lines: (**A**) Western blot validation of CD73 knockout at the protein level; (**B**) Expression of CD73 mRNAs in monoclonal cells and control cells (WT); **** *p* < 0.0001; (**C**) Staggered offset showing the expression levels of CD73 on monoclonal cells and control cells via flow cytometry. The uncropped blots are shown in Figure S1.

To verify the role of CD73 in pancreatic cancer cell lines, one human cell line (PANC1) and one murine cell line (TB32047) were used to perform a CRISPR/Cas9-based knockout of CD73. Two sgRNAs targeting different regions of the human and murine CD73 exons, respectively, were designed, and CRISPR/Cas9 gene editing was performed to introduce mutations into this region. We verified the knockout of CD73 at the protein level using Western blot analysis (Figure 2A). The results showed no CD73 protein level expression in the monoclonal group. The transcription of CD73 mRNAs was inhibited, and this was verified via qRT-PCR (Figure 2B). The expression of CD73 mRNAs in single clones of TB32047 and PANC1 was significantly downregulated, as compared to controls. The expression of CD73 on the cell membrane’s surface was verified via flow cytometry (Figure 2C). CD73 knockout resulted in essentially no expression on the cell membrane’s surface. The mutations of genomic DNA in single clones of both TB32047 cells and PANC1 cells were confirmed via DNA sequencing (Table S6).

### 3.3. CD73 Knockout Inhibits Cell Proliferation and Induces G1 Phase Arrest with No Effect on Apoptosis

**Figure 3:**
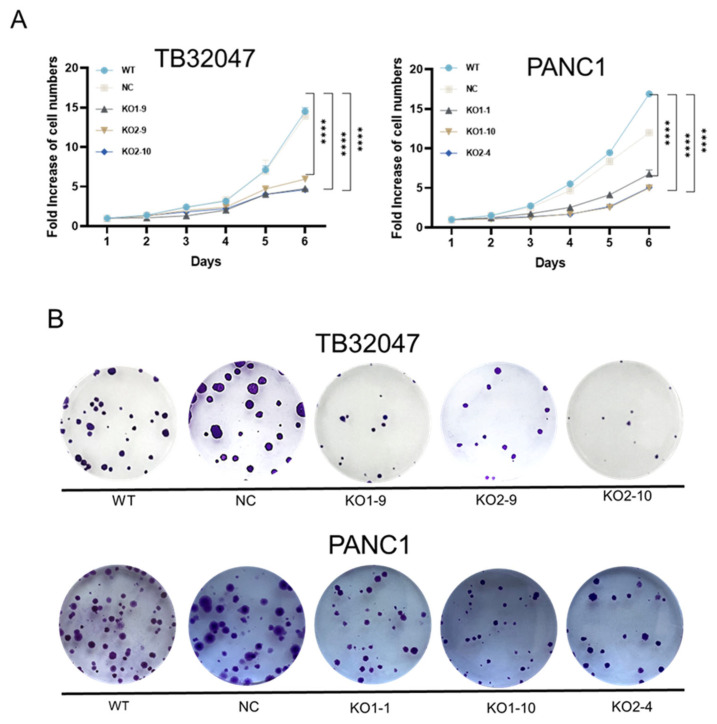

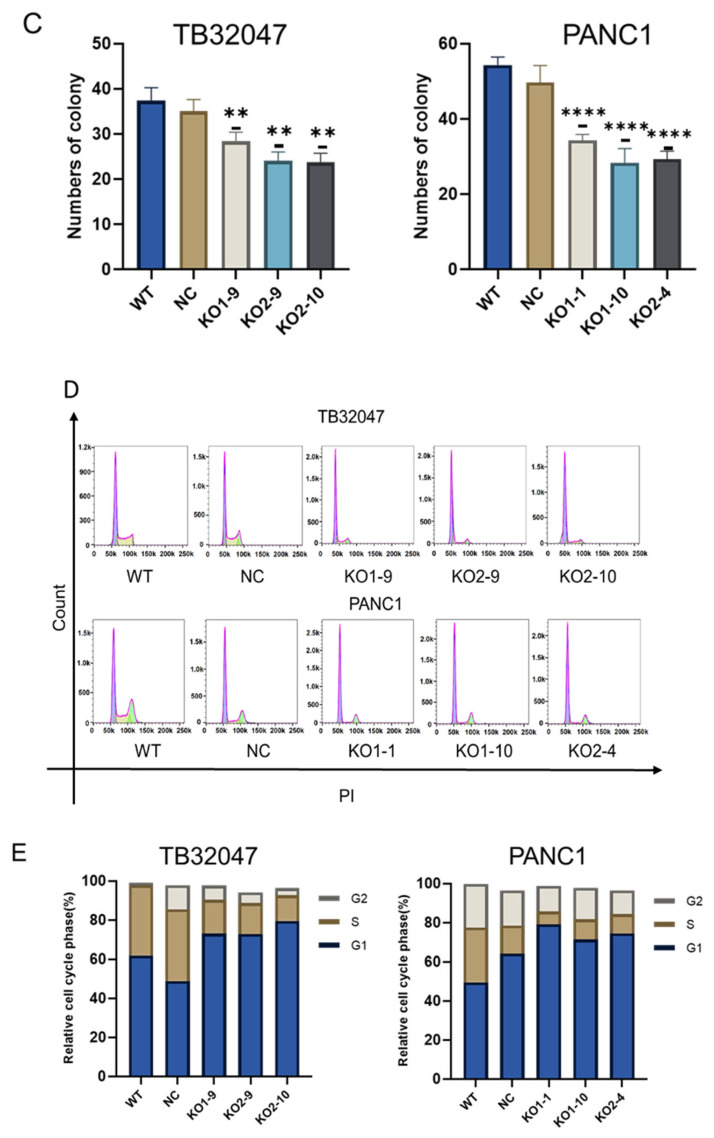
Loss of CD73 inhibited cell growth and blocked the G1 phase of the cell cycle in pancreatic cancer: (**A**) Effects of CD73 knockout on the growth of pancreatic cancer cells in vitro. Cell proliferation assays were performed for 6 days. Data are expressed as the mean ± SEM of three experiments; (**B**,**C**) The colony formation assays of TB32047 and PANC1 cells were performed after knockout of CD73. The colony numbers were counted, and data were presented as means of three independent experiments (*n* = 3); ** *p* < 0.01 and **** *p* < 0.0001; (**D**) Cell cycle analysis of TB32047 and PANC1 cells. Representative figures from three independent experiments (*n* = 3) are shown; (**E**) Histogram of the percentage of G1, S, and G2 phases in each cell.

TB32047 (WT, NC, KO1-9, KO2-9, and KO2-10) and PANC1 (WT, NC, KO1-1, KO1-10, and KO2-4) cells were seeded at 1000 cells per well in black 96-well plates and incubated for 6 days. All cells exhibited robust logarithmic growth under these conditions until fully confluent. After the loss of CD73 in TB32047 and PANC1 cells, cell growth was significantly inhibited, as compared with the wild-type cells (Figure 3A). Clonogenic assays showed a dramatic decrease in the colony numbers of the TB32047 and PANC1 KOs after knockout of CD73, as compared to those of the WT and NC cells (Figure 3B,C). 

Cell cycle detection via flow cytometry found that knockout of CD73 induced G1 phase arrest (Figure 3D). For the TB32047 cell line, the G1 phase of KO1-9, KO2-9, and KO2-10 cells accounted for 73.0%, 72.8%, and 75.9% of the entire cell cycle, respectively. At the same time, WT and NC cells’ G1 phases were found to be 61.9% and 48.9%. For the PANC1 cell line, the G1 phase of KO1-1, KO1-10, and KO2-4 cells accounted for 79.1%, 71.4%, and 74.5% of the entire cell cycle, respectively; WT and NC cells’ G1 phases are 49.5% and 64.1% (Figure 3E). We also detected the effect on apoptosis after knockout of CD73, and the results show no significant change between the knockout group and the control group (Figure S1).

### 3.4. CD73 Knockout Inhibits Cell Migration In Vitro

**Figure 4:**
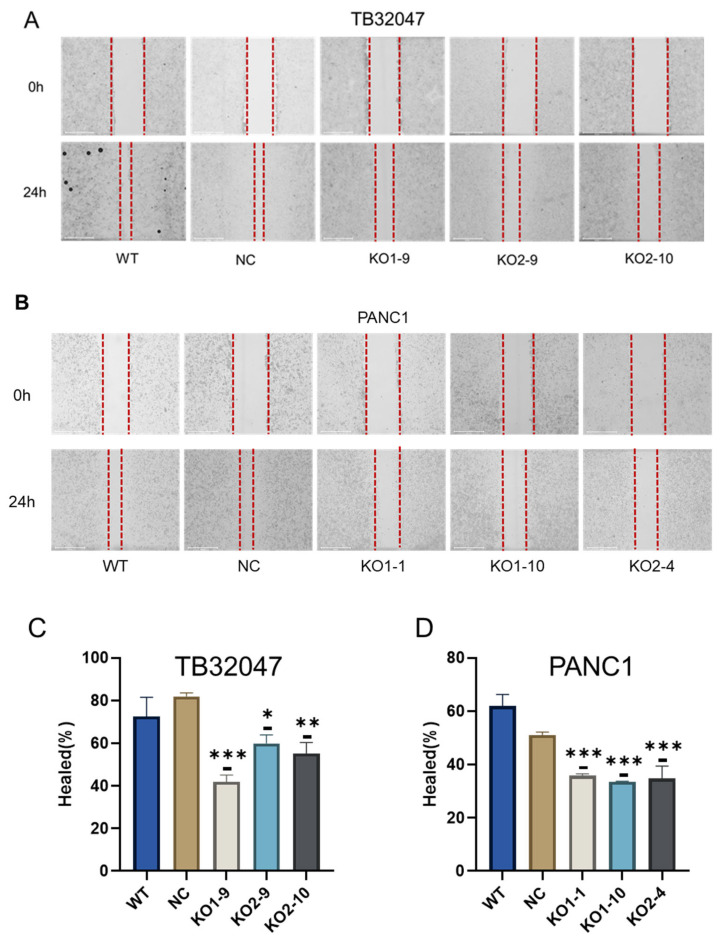
CD73 knockout inhibits pancreatic cancer cell migration: (**A**,**B**) Representative images show the area covered by the TB32047 and PANC1 cells at 0 h and 24 h after wounding; dotted lines represents the edge of cell growth (**C**,**D**) Percentage of cell migration was determined by the rate of cells moving towards the scratched area over time as calculated with ImageJ ™ software; data were presented as means of three independent experiments (*n* = 3); * *p* < 0.05, ** *p* < 0.01, and *** *p* < 0.001.

With the wound healing assay, we showed that the knockout of CD73 significantly inhibits the migration of PDAC cells (Figure 4A,B). For the TB32047 cell line, KO1-9, KO2-9, and KO2-10 healed 41.98%, 59.95%, and 55.18% of the wound area after 24 h, while WT and NC cells healed more than 75% of the wound area (Figure 4C). For the PANC1 cell line, KO1-1, KO1-10, and KO2-4 healed 37.77%, 32.18%, and 33.36% of the wound area after 24 h, but the control cells healed more than 55% of the wound area (Figure 4D).

### 3.5. CD73 Deficiency Inhibits the Phosphorylation of ERK and STAT3 in Pancreatic Cancer, and It Promotes E-Cadherin Expression in TB32047 Cells

**Figure 5:**
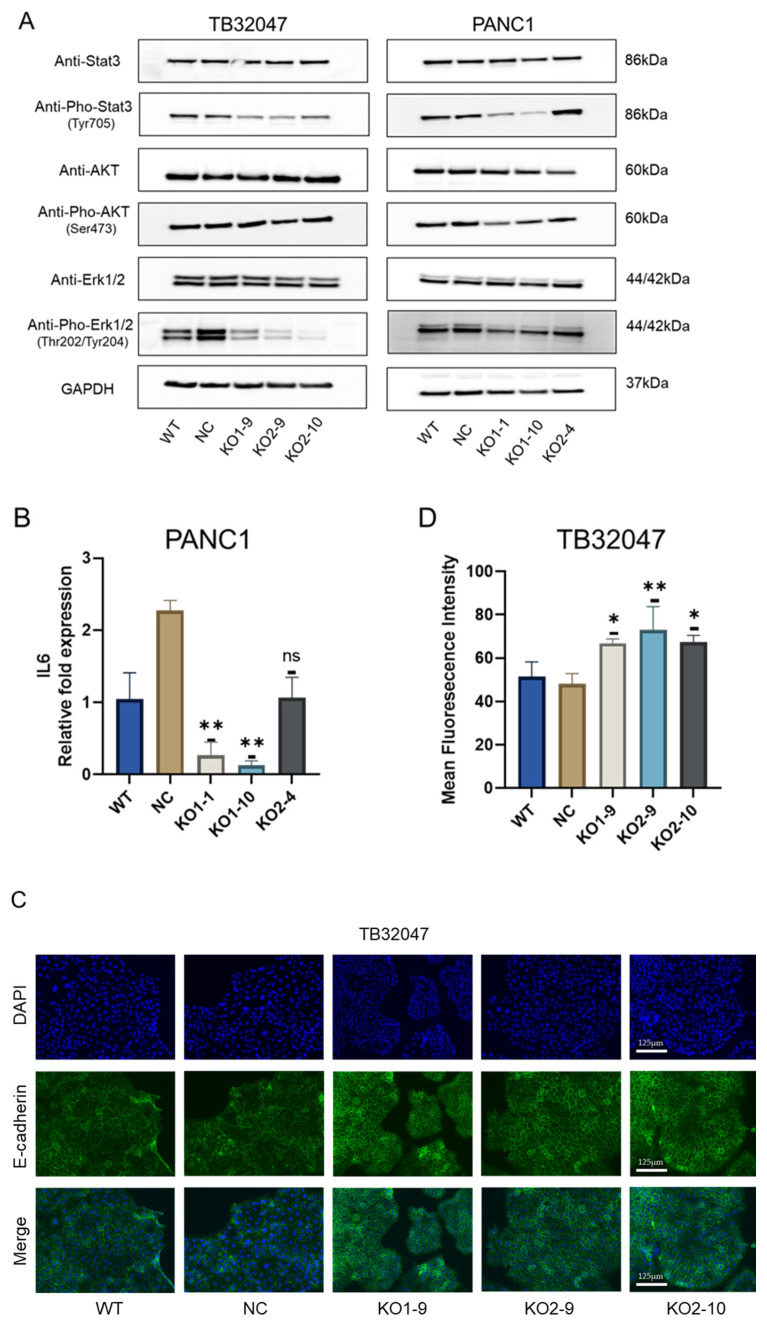
Knockout of CD73 inhibits phosphorylation of ERK and STAT3 and promotes E-cadherin expression: (**A**) Phosphorylation of ERK and STAT3 was downregulated in TB32047 and PANC1 cells after CD73 knockout; (**B**) IL-6 expression was decreased in PANC1 (KO1-1, KO1-10) cells; (**C**) Immunofluorescence staining of combined E-cadherin (green), DAPI (blue), and TB32047-KOs and controls (WT and NC cells were transfected with a non targeting sgRNA); enhanced expression of monoclonal E-cadherin; Scale bar = 125 μm (**D**) Mean immunofluorescence intensity in TB-KOs and controls. Bars represent the mean values and the corresponding standard errors of the mean (SEMs) of *n* = 3 independent experiments. Significance: * *p*  <  0.05 and ** *p*  <  0.01, ns means no significance. The uncropped blots are shown in Supplementary File S1.

After the knockout of CD73 in TB32047 and PANC1 cells, we observed a significant reduction in the phosphorylation of ERK (intracellular mononuclear regulatory kinase). Phosphorylation of STAT3 (signal transducer and activator of transcription 3) was also significantly reduced in TB32047 cells, while two monoclonal clones (KO1-1 and KO1-10) in PANC1 showed low levels of expression. However, no significant change in Akt was observed (Figure 5A). In addition, positive feedback of CD73 with IL-6 (interleukin 6) has been reported in human breast cancer [25]. We verified in PANC1 (KO1-1 and KO1-10) cells that knockout of CD73 reduced phosphorylation of STAT3 and affected IL-6 expression simultaneously (Figure 5B). We also found that CD73 knockout prompted increased E-cadherin expression in TB32047 cells, as detected by immunofluorescence (Figure 5C,D). In contrast, no significant changes were observed in PANC1 cells (Figure S2).

### 3.6. CRISPR/Cas9 Screen of Inducible Regulators of CD73 Expression in Pancreatic Cancer Cells

**Figure 6:**
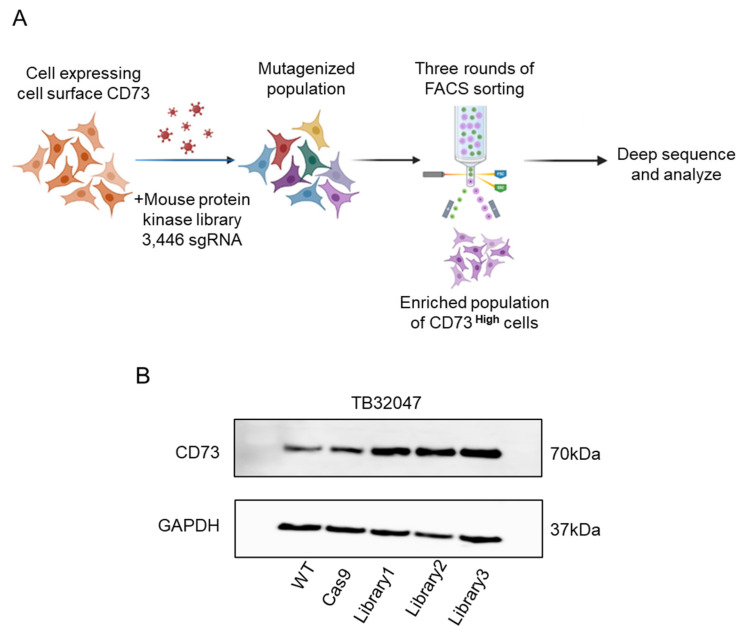
Schematic diagram of flow cytometry sorting and results: (**A**) Schematic diagram of the timeline and experimental procedure for CRISPR/Cas9 screening using the protein kinase library; (**B**) Western blot analysis was performed to verify the protein expression level of CD73 after 3 rounds of sorting. The uncropped blots are shown in Supplementary File S1.

To identify genes that regulate CD73 expression, we used CRISPR/Cas9 loss-of-function screening combined with FACS technology to establish a screening model in the mouse pancreatic cancer cell line TB32047 (Figure 6A). We used protein-kinase-based screens to determine which gene can regulate CD73 expression. A total of 3446 sgRNAs targeting 915 genes were introduced into TB32047-Cas9 cells at a multiplicity of infection (MOI) of 0.3. Then, the transfected cells were screened for high levels of CD73 expression. As negative controls, the sgRNAs targeting CD73 are present in our protein kinase library. After the first round of sorting, we continued to culture the cells to guarantee enough read-death and coverage for the next round of screening. After three rounds of enrichment, we obtained three groups of CD73 high-expression cells (Figure 6B). Associated sgRNAs were identified in the CD73 high-expression group by deep sequencing.

**Figure 7:**
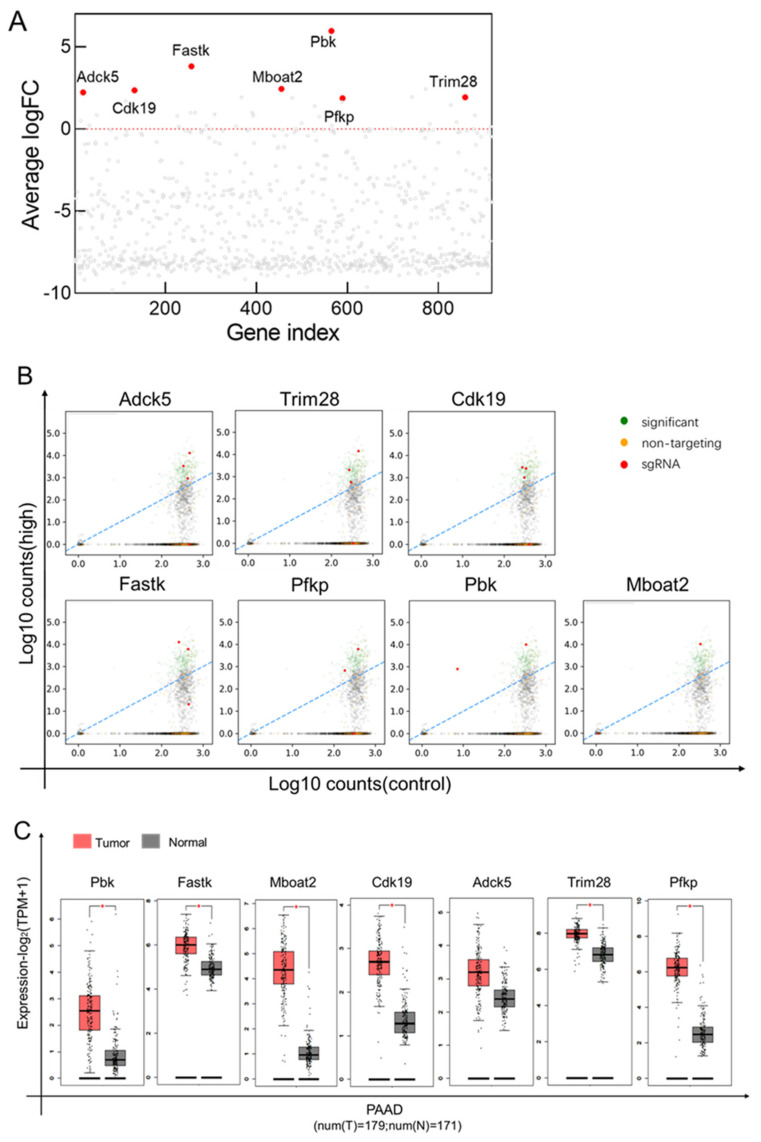
Analysis of the deep sequence results of the CD73 high-expression group: (**A**) The graph shows the enrichment of candidate genes (red dots) in the CD73 high-expression group, as identified by PinAPL-Py, versus other genes in the library (gray dots) after selection; The red dotted lines represent the cutoff for the average LogFC is 0. (**B**) PinAPL-Py analysis produced sgRNA enrichment maps for each highly expressed gene; Scatterplots showing log10 (normalized) read counts for each sgRNA from the treatment sample (*y*-axis) versus control sample’s average (*x*-axis), sgRNAs yielding significant enrichment marked in green. The red represents the target sgRNA, and the name is above the picture. (**C**) The TCGA (PAAD) and GTEx (PAAD) databases obtained from GEPIA2 showed that these genes were differentially expressed in the tissue of pancreatic cancer tumors, as compared to normal tissue. * *p*  <  0.05.

PinAPL-Py was used to analyze the deep sequencing results for the CD73 high-expression group. We found that seven sgRNAs (Pbk, Fastk, Cdk19, Adck5, Trim28, and Pfkp) were significantly increased in the CD73 high-expression group, as compared with untreated cells (Figure 7A). Adck5, Trim28, and Cdk19 sgRNA-enrichment levels were relatively high (Figure 7B). This indicates that these sgRNA-targeted genes may be potential regulators of CD73 for increasing its expression. In addition, the TCGA (PAAD) and GTEx (PAAD) databases obtained via GEPIA2 showed that these genes were expressed at higher rates in the tissues of pancreatic cancer tumors than in normal tissues, with the exception of Adck5 (Figure 7C).

## 4. Discussion

Although some patients with early-stage pancreatic cancer can undergo radical resection, most patients with advanced disease have already lost their chance for surgery [26]. Immune checkpoint therapies for tumors have received increasing amounts of attention in recent years. Many of these immune checkpoints are CD molecules [27,28,29]. These CD molecules are involved in cancer development and play an essential role in the treatment and prognosis of cancer. Flow cytometry was used to test the expression of various CD molecules in pancreatic cancer cells in this study. We determined that the relative expression of CD73 is high in TB32047 and PANC1 cell membranes, and this was further validated using qRT-PCR. In addition, we clarified that CD73 is significantly differentially expressed in pancreatic cancer and normal pancreatic tissues using GEPIA analysis. However, high expression levels of CD73 in cancer tend to promote tumor progression [20]. Therefore, the targeted blockade of CD73 may be a beneficial therapeutic approach for treating cancer patients in the future.

The detailed functions of CD73 in pancreatic tumor cells and the genes affecting its expression remain to be determined. In this study, CRISPR/Cas9 gene-editing technology was applied to inactivate CD73. The exons of CD73 were edited at the indicated positions by the specific binding of sgRNA-guided Cas9 nuclease and genomic DNA, which was confirmed by genomic DNA sequencing. The alteration of base sequences between exons decreases mRNA transcription efficiency, and qRT-PCR confirmed that CD73 mRNA expression was significantly repressed. Western blot analysis verified the knockout of CD73 at the protein level. Knockout of CD73 on the cell membrane’s surface was verified by flow cytometry.

We found that the total knockout of CD73 significantly inhibits cell proliferation in TB32047 and PANC1 cells, and cell cycle G1 is blocked. This result is consistent with Zhou, L. et al. [30]. However, we did not observe any effect on apoptosis after the knockout of CD73. We also found that the knockout of CD73 decreases cell migration ability. 

Extracellular signal-regulated kinases (ERK) are widely expressed protein kinase intracellular signaling molecules involved in functions including cell proliferation, cell growth, cell metabolism, and cell migration [31,32]. Knockout of CD73 in pancreatic cancer significantly inhibits ERK activation. Some studies have investigated the induction of G1 phase arrest via the AKT/ERK/cyclin D signaling pathway after CD73 knockdown [30]. However, our study found no significant changes in AKT.

STAT3 (signal transducer and activator of transcription 3) is a protein-coding gene member of the STAT protein family [33]. STAT3 is highly expressed in many different types of cancers [34]. It is highly expressed in breast cancer, and it promotes cancer proliferation and migration [35]. The key role of STAT3 in promoting the progression of pancreatic cancer is well-established, but the way to inhibit STAT3 activity remains to be investigated [36]. We found reduced phospho-STAT3 expression in TB32047 monoclonal cells and in PANC1 KO1-1 and KO1-10 cells. This suggests that CD73 may be one of the proteins that inhibits STAT3 activity in pancreatic cancer. We also found that PANC1 KO1-1 and KO1-10 cells inhibited the expression of interleukin-6 (IL-6). The IL-6/JAK/STAT3 pathway is aberrantly over-activated in many types of cancer and drives tumor cell proliferation, survival, invasion, and metastasis, while it strongly suppresses antitumor immune responses [37]. Therefore, we suspect that in pancreatic cancer, knockout of CD73 may lead to the inhibition of the IL-6/JAK/STAT3 pathway, thus inhibiting tumor cell growth. This result was partially validated in the PANC1 cell line.

E-cadherin is a tumor-suppressor gene [38]. E-cadherin has been studied more in pancreatic cancer and is mainly associated with the invasion and metastasis of pancreatic cancer cells [39]. Targeting the expression of E-cadherin with small-molecule drugs is a new way to treat GI cancers [40]. While the genes regulating E-cadherin expression in pancreatic cancer are still unclear, we found that the expression of E-cadherin was significantly increased in TB32047 cells after knockout of CD73. This suggests that the high expression of CD73 in pancreatic cancer may inhibit the E-cadherin pathway and thus promote tumor migration.

We identified candidate genes regulating CD73 using an sgRNA library focused on kinase- and PDAC-related genes in the KPC-mouse-model-derived cell line TB32047 using a systematic approach of reverse selection using CRISPR/Cas9. Pbk, Fastk, Cdk19, Adck5, Trim28, and Pfkp may be the regulatory genes for CD73 in pancreatic cancer. Pbk (PDZ Binding Kinase) is a Protein Coding gene. Pbk has been shown to be an essential gene in the regulation of mitosis and tumorigenesis, but the role of PBK in various cancers remains unclear [41]. Fas-activated serine/threonine kinase is an enzyme that, in humans, is encoded by the FASTK gene [42]. CDK19 (Cyclin Dependent Kinase 19) is a Protein Coding gene. CDK19 is associated with increased aggressiveness and shorter disease-free survival in primary prostate cancer [43]. ADCK5 belongs to the protein kinase superfamily. The function of this protein is not yet clear. It has been shown that ADCK5 regulates lung cancer cell invasion and migration. It regulates the expression of the oncogene human pituitary tumor transforming gene-1 (PTTG1) by phosphorylating the transcription factor SOX9, which enhances the migration and invasion of lung cancer cells [44]. Tripartite motif-containing 28 (TRIM28), also known as transcriptional intermediary factor 1β (TIF1β) and KAP1 (KRAB-associated protein-1), is a protein that, in humans, is encoded by the TRIM28 gene [45]. Pfkp is an enzyme that, in humans, is encoded by the PFKP gene. Pfkp plays a critical role in many steps of cancer initiation and metastasis [46]. Although we need more studies to confirm the precise regulation of CD73, the list of post-selected genes obtained thus far provides a good entry point.

In this study, we performed multiple screens with flow cytometry and selected the top 3% of high-CD73-expressing cells in each screen. By analyzing the deep sequencing results, we found that a large amount of sgRNA was lost in the screening due to overly stringent screening and differences in transfection efficiency between groups, which may have affected the screening results. Therefore, we suggest that the screening conditions be relaxed to 5–10%. In addition, CRISPR/Cas9, as a gene-editing tool, has considerable off-target effects that can affect the experimental results. When sgRNA (a short-stranded RNA that matches a target DNA fragment) directs CRISPR/Cas9 to edit the genome, it promotes undesired, off-target mutagenesis because sgRNA can tolerate certain mismatches with DNA targets. Therefore, as much as possible, we needed to perform multiple transfection sequencing to rule out false-positive results due to off-target effects. Recent studies have shown that genetic and behavioral characteristics vary significantly between the cell lines of different origins and subtypes, even within pancreatic cancer cell lines [47]. We also observed that the pathways affecting CD73 are not identical in murine- and human-lineage pancreatic cancer cells, so it would be prudent to continue crossover or combinatorial screening between different cell lines and different species in future experiments to find the critical targets for regulating CD73.

## 5. Conclusions

We verified that CD73 expression is increased in pancreatic cancer, that knockout of CD73 inhibits cell proliferation and migration, and that it blocks the G1 phase of the cell cycle. We also found that the deletion of CD73 inhibits the ERK/STAT3 pathway and activates the E-cadherin pathway. Pbk, Fastk, Cdk19, Adck5, Trim28, and Pfkp may be the regulatory genes for CD73 in pancreatic cancer.

## Data Availability

The data presented in this study are available in the article and in the Supplementary Materials.

## Supplementary Materials

The following supporting information can be downloaded at: https://www.mdpi.com/article/10.3390/cancers15194842/s1, Figure S1: Knockout of CD73 did not affect cell apoptosis; Figure S2: Immunofluorescence staining of combined E-cadherin (green), DAPI (blue), and PANC1-KOs and controls; Table S1: List of antibodies for FACS; Table S2: List of plasmids; Table S3: List of primers for the single-clone check; Table S4: List of qRT-PCR primers; Table S5: List of antibodies for Western blot analysis; Table S6: Sequencing confirmation of mutations in TB32047- and PANC1-CD73-knockout single clones. Supplementary File S1: Western Blot raw data and Image densitometry.
