# Supplementary material for: Impact of CRISPR/Cas9-Mediated CD73 Knockout in Pancreatic Cancer
Source: Cancers (Basel). 2023 Oct 3;15(19):4842. doi: 10.3390/cancers15194842 (PMC10572021; doi:10.3390/cancers15194842)
Supplement: Supplementary file 1 [file cancers-15-04842-s001.zip › Supporting document1/Table S1 List of antibodies for FACS.pdf]

**BD**	CD73	Human	Cat# 561014<br>RRID:AB_2033967	PE
	CD80	Human	Cat# 564158<br>RRID:AB_2738630	BV650
	CD154	Human	Cat# 566268<br>RRID:AB_2739646	BV421
	CD252	Human	Cat# 563766<br>RRID:AB_2738412	BV421
	CD276	Human	Cat# 565829<br>RRID:AB_2739369	BV421
	CD73	Maus	Cat# 550741<br>RRID:AB_393860	PE
	CD80	Maus	Cat# 563687<br>RRID:AB_2738376	BV650
	CD154	Maus	Cat# 561719<br>RRID:AB_10897018	PE
	CD252	Maus	Cat# 565341<br>RRID:AB_2739194	BV421
	CD276	Maus	Cat# 563634<br>RRID:AB_2738336	BV421
