# Supplementary material for: Impact of CRISPR/Cas9-Mediated CD73 Knockout in Pancreatic Cancer
Source: Cancers (Basel). 2023 Oct 3;15(19):4842. doi: 10.3390/cancers15194842 (PMC10572021; doi:10.3390/cancers15194842)
Supplement: Supplementary file 1 [file cancers-15-04842-s001.zip › Supporting document1/Table S3 List of primers for the single-clone check.pdf]

sgRNA	Direction	Sequences (5'->3')
Ho_CD73_sg1_check	Forward	ACCAGCGAGGACTCCAGCAA
Ho_CD73_sg1_check	Reverse	AATCGTCCAAGGGACTTCTATGC
Ho_CD73_sg2_check	Forward	TTTGGTTTTACTGACTCTTGAGC
Ho_CD73_sg2_check	Reverse	ATCCTTTTGAAACAATTACCTGTG
Mm_CD73_sg1_check	Forward	CCTTTCCTCCCTCCCTAGACGC
Mm_CD73_sg1_check	Reverse	GCCACAACCAAAATGCACAGATG
Mm_CD73_sg2_check	Forward	TACTTAGGCACTGGGAAATCAT
Mm_CD73_sg2_check	Reverse	ACAAAGAAGTTCACCGAGCAGA
