# Supplementary material for: Impact of CRISPR/Cas9-Mediated CD73 Knockout in Pancreatic Cancer
Source: Cancers (Basel). 2023 Oct 3;15(19):4842. doi: 10.3390/cancers15194842 (PMC10572021; doi:10.3390/cancers15194842)
Supplement: Supplementary file 1 [file cancers-15-04842-s001.zip › Supporting document1/Western Blot raw data and Imaje densitometry.pdf]

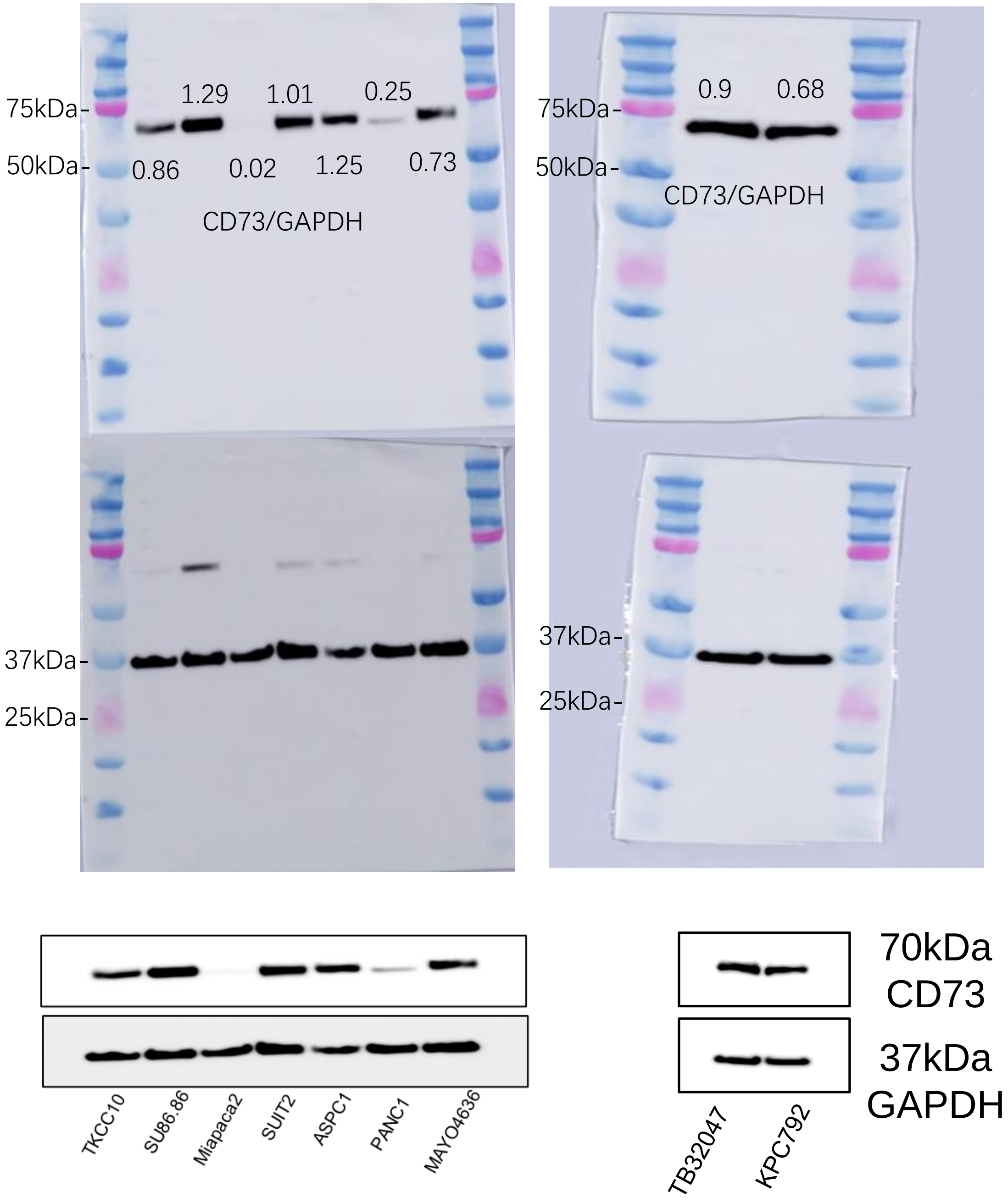
75kDa
50kDa
1.29
1.01
0.25
0.86
0.02
1.25
0.73
CD73/GAPDH
0.9
0.68
CD73/GAPDH
37kDa
25kDa
37kDa
25kDa
70kDa
CD73
37kDa
GAPDH
TKCC10
SU86.86
Miapaca2
SUIT2
ASPC1
PANC1
MAYO4636
TB32047
KPC792

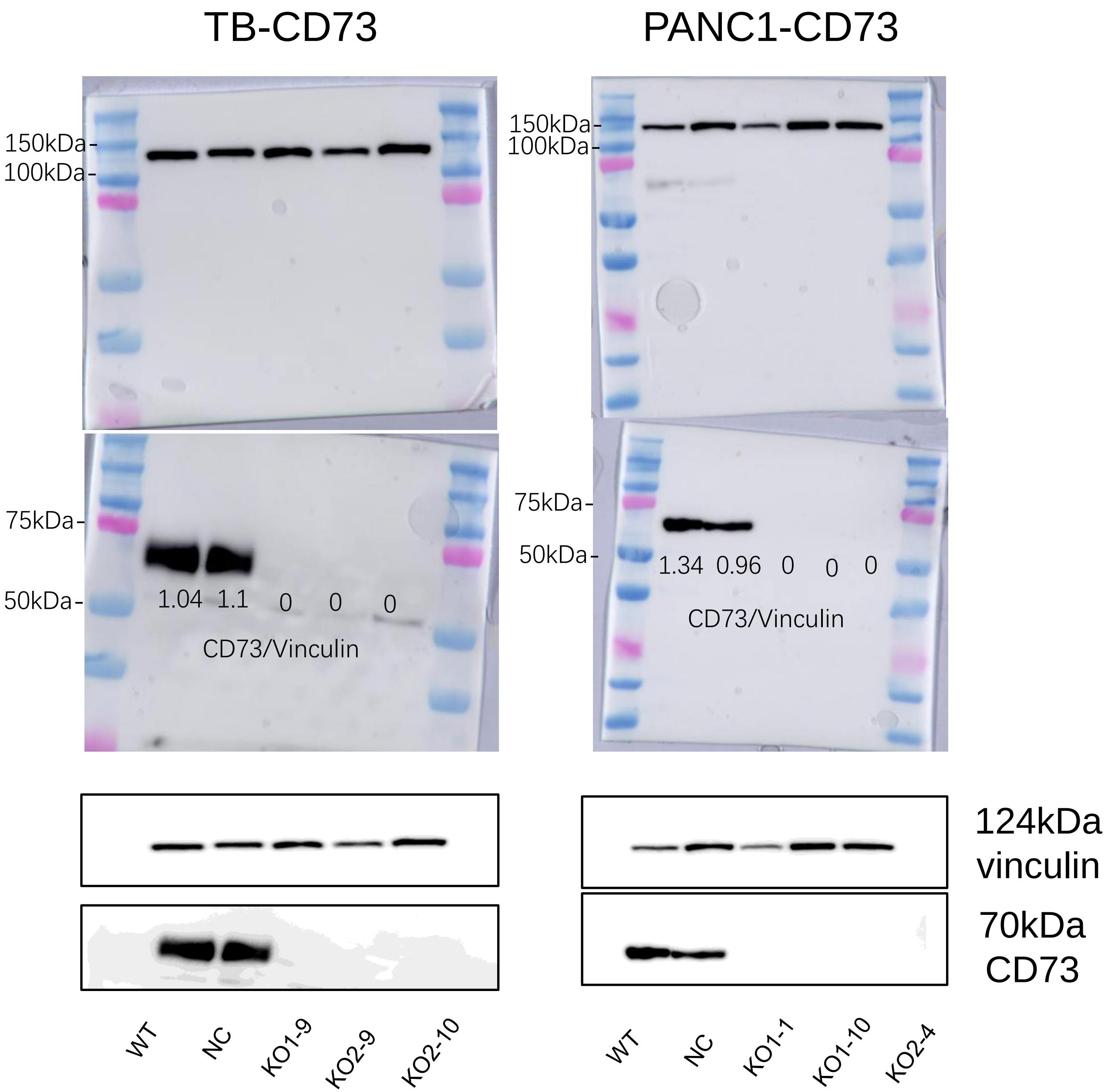

TB-CD73
PANC1-CD73
150kDa
100kDa
150kDa
100kDa
75kDa
50kDa
1.04
1.1
0
0
0
CD73/Vinculin
75kDa
50kDa
1.34
0.96
0
0
0
CD73/Vinculin
124kDa
vinculin
70kDa
CD73
WT
NC
KO1-9
KO2-9
KO2-10
WT
NC
KO1-1
KO1-10
KO2-4

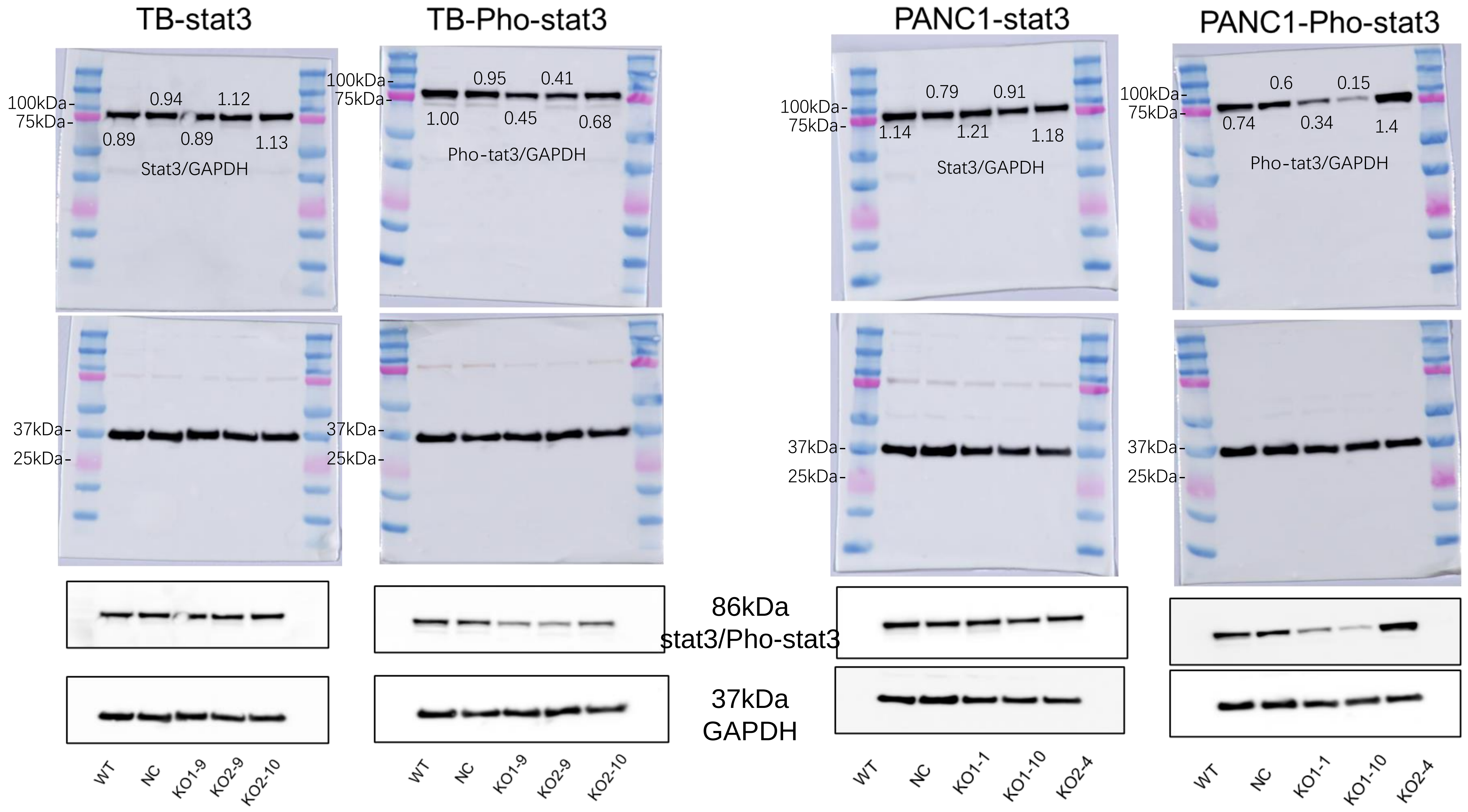
TB-stat3
TB-Pho-stat3
PANC1-stat3
PANC1-Pho-stat3
100kDa-
75kDa-
0.94
1.12
0.89
0.89
1.13
Stat3/GAPDH
0.95
0.41
1.00
0.45
0.68
Pho-tat3/GAPDH
0.79
0.91
1.14
1.21
1.18
Stat3/GAPDH
0.6
0.15
0.74
0.34
1.4
Pho-tat3/GAPDH
37kDa-
25kDa-
86kDa
stat3/Pho-stat3
37kDa
GAPDH
WT
NC
KO1-9
KO2-9
KO2-10
WT
NC
KO1-9
KO2-9
KO2-10
WT
NC
KO1-1
KO1-10
KO2-4
WT
NC
KO1-1
KO1-10
KO2-4

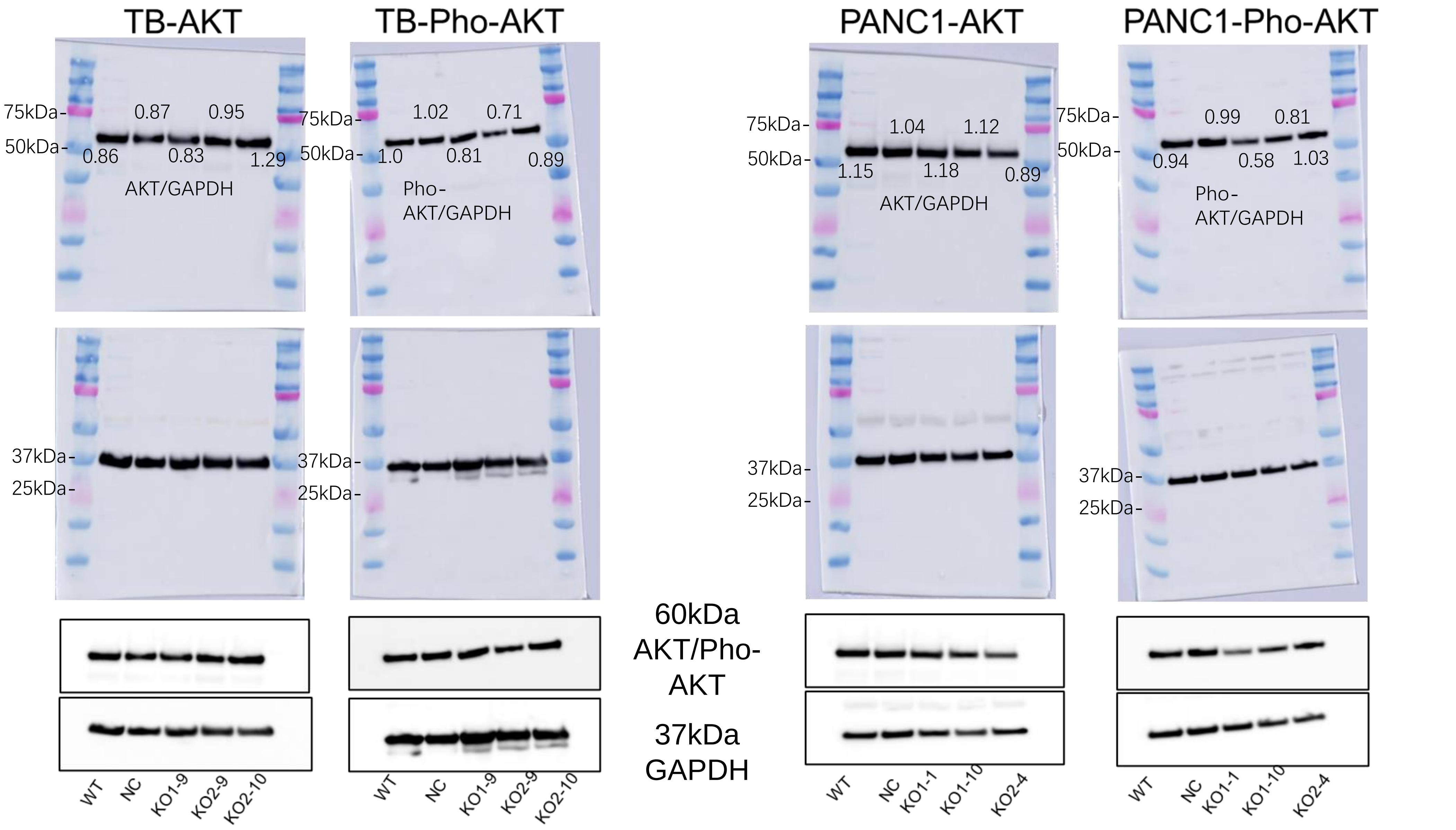
TB-AKT
TB-Pho-AKT
PANC1-AKT
PANC1-Pho-AKT
75kDa-
50kDa-
37kDa-
25kDa-
0.86
0.87
0.83
0.95
1.29
AKT/GAPDH
1.0
1.02
0.81
0.71
0.89
Pho-AKT/GAPDH
1.15
1.04
1.18
1.12
0.89
AKT/GAPDH
0.94
0.99
0.58
0.81
1.03
Pho-AKT/GAPDH
60kDa AKT/Pho-AKT
37kDa GAPDH
WT
NC
KO1-9
KO2-9
KO2-10
WT
NC
KO1-1
KO1-10
KO2-4

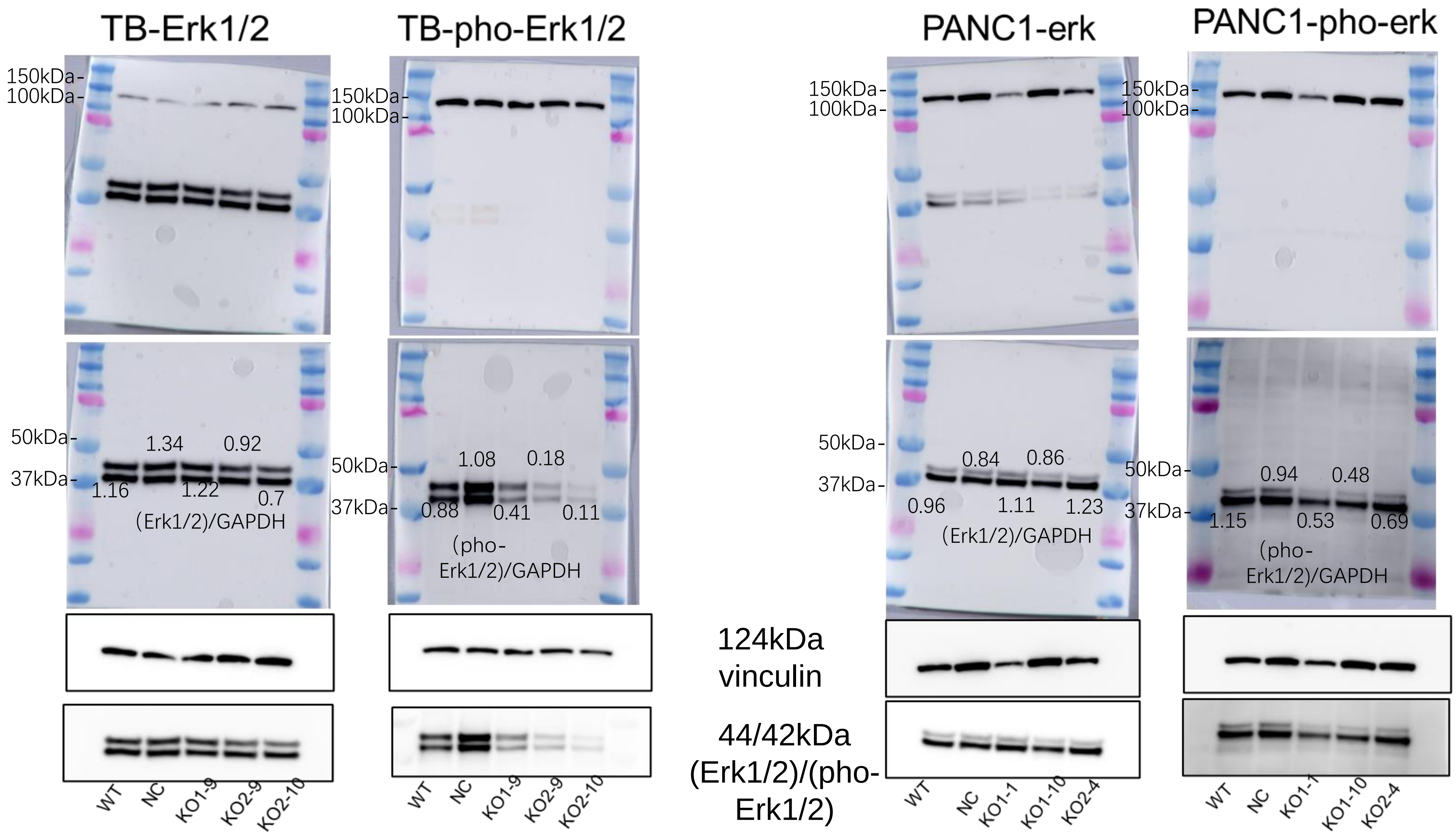
TB-Erk1/2
TB-pho-Erk1/2
PANC1-erk
PANC1-pho-erk
150kDa
100kDa
50kDa
37kDa
1.34
0.92
1.16
1.22
0.7
(Erk1/2)/GAPDH
1.08
0.18
0.88
0.41
0.11
(pho-Erk1/2)/GAPDH
0.84
0.86
0.96
1.11
1.23
(Erk1/2)/GAPDH
0.94
0.48
1.15
0.53
0.69
(pho-Erk1/2)/GAPDH
124kDa vinculin
44/42kDa (Erk1/2)/(pho-Erk1/2)
WT
NC
KO1-9
KO2-9
KO2-10
WT
NC
KO1-1
KO1-10
KO2-4

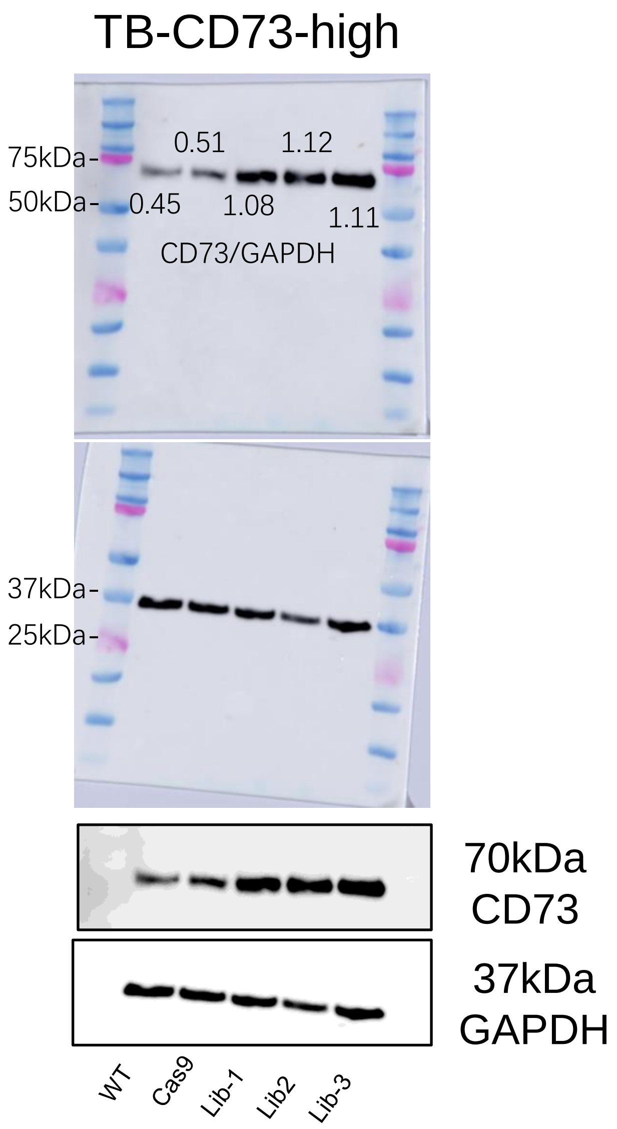
TB-CD73-high
75kDa-
50kDa-
0.51
1.12
0.45
1.08
1.11
CD73/GAPDH
37kDa-
25kDa-
70kDa
CD73
37kDa
GAPDH
WT
Cas9
Lib-1
Lib2
Lib-3
