# Supplementary material for: Impact of CRISPR/Cas9-Mediated CD73 Knockout in Pancreatic Cancer
Source: Cancers (Basel). 2023 Oct 3;15(19):4842. doi: 10.3390/cancers15194842 (PMC10572021; doi:10.3390/cancers15194842)
Supplement: Supplementary file 1 [file cancers-15-04842-s001.zip › Supporting document1/Table S4 List of qRT-PCR primers.pdf]

**Primer target**	**Direction**	**Sequences (5'->3')**
Ho_CD73	Forward	CCAGTACCAGGGCACTATCTG
Ho_CD73	Reverse	TGGCTCGATCAGTCCTTCCA
Ho_CD80	Forward	GGCCCGAGTACAAGAACCG
Ho_CD80	Reverse	TCGTATGTGCCCTCGTCAGAT
Ho_CD154	Forward	ACATACAACCAAACTTCTCCCCG
Ho_CD154	Reverse	GCAAAAAGTGCTGACCCAATCA
Ho_CD252	Forward	GGTCAGGTCTGTCAACTCCTT
Ho_CD252	Reverse	CATCCAGGGAGGTATTGTCAGT
Ho_CD276	Forward	TCACAGGGCAGCCTATGAC
Ho_CD276	Reverse	TCCTCAGCTCCTGCATTCTC
Mm_CD73	Forward	ATGCCGGAGACCAGTACCA
Mm_CD73	Reverse	CAGTGCCATAGCATCGTAGCC
Mm_CD80	Forward	ACCCCCAACATAACTGAGTCT
Mm_CD80	Reverse	TTCCAACCAAGAGAAGCGAGG
Mm_CD154	Forward	CTTCTGCTCTAATCGGGAGCC
Mm_CD154	Reverse	GCCGCCTTGAGTAAGATTCTC
Mm_CD276	Forward	GGACCTACGTCCAGGGAACAT
Mm_CD276	Reverse	TGGTCACATTGCCAGTCAAGG
Ho_GAPDH	Forward	CTTTGGTATCGTGGAAGGACTC
Ho_GAPDH	Reverse	AGTAGAGGCAGGGATGATGT
Ho_β-Actin	Forward	CACCATTGGCAATGAGCGGTTC
Ho_β-Actin	Reverse	AGGTCTTTGCGGATGTCCACGT
Mm_GAPDH	Forward	AGGTCGGTGTGAACGGATTTG
Mm_GAPDH	Reverse	TGTAGACCATGTAGTTGAGGTCA
Mm_β-Actin	Forward	GTGACGTTGACATCCGTAAAGA
Mm_β-Actin	Reverse	GCCGGACTCATCGTACTC
