# Supplementary material for: Impact of CRISPR/Cas9-Mediated CD73 Knockout in Pancreatic Cancer
Source: Cancers (Basel). 2023 Oct 3;15(19):4842. doi: 10.3390/cancers15194842 (PMC10572021; doi:10.3390/cancers15194842)
Supplement: Supplementary file 1 [file cancers-15-04842-s001.zip › Supporting document1/Table S5 List of antibodies for Western blot analysis.pdf]

Source	Antibodies	Species	Identifier	Note
**Cell signaling**	NT5E/CD73	Human, Maus	Cat# 13160 RRID:AB_2716625	70 kDa
	Vinculin	Human, Maus	Cat# 13901 RRID:AB_2728768	124 kDa
	GAPDH	Human, Maus	Cat# 5174 RRID:AB_10622025	37 kDa
	P44/42 MAPK(Erk1/2)	Human, Maus	Cat# 4695 RRID:AB_390779	44/42 kDa
	AKT-pan	Human, Maus	Cat# 4691 RRID:AB_915783	60 kDa
	Stat3	Human, Maus	Cat# 12640 RRID:AB_2629499	86 kDa
	p-AKT	Human, Maus	Cat# 4060 RRID:AB_2315049	60 kDa
	p-Stat3	Human, Maus	Cat# 9145 RRID:AB_2491009	86 kDa
	Phospho-p44/42 MAPK(p-Erk1/2)	Human, Maus	Cat# 4370 RRID:AB_2315112	44/42 kDa
	E-Cadherin	Human, Maus	Ca#3195 RRID:AB_2291471	135 kDa
	HRP-linked anti-rabbit IgG	Human, Maus	Cat# 7074 RRID:AB_2099233	/
	HRP-linked anti-mouse IgG	Human, Maus	Cat# 7076 RRID:AB_330924	/
