# Supplementary material for: Impact of CRISPR/Cas9-Mediated CD73 Knockout in Pancreatic Cancer
Source: Cancers (Basel). 2023 Oct 3;15(19):4842. doi: 10.3390/cancers15194842 (PMC10572021; doi:10.3390/cancers15194842)
Supplement: Supplementary file 1 [file cancers-15-04842-s001.zip › Supporting document1/Table S6 Sequencing confirmation of mutations in TB32047- and PANC1-CD73-knockout single clones.[176].pdf]

TB32047 Clones	Indel Size	PANC1 Clones	Indel Size
1-9	Delete 1bp	1-1	Delete 5 bp
2-9	Insert 1bp/ Delete 92bp	1-10	Insert 265bp
2-10	Delete 2bp/ Delete 26 bp	2-4	Delete 11 bp

	TB32047-WT or NC<br>NC_000075.7 37155-37174bp	
5'-	GAAGCGGCACGTCTGAGTGG	-3'
3'-	CTTCGCCGTGCAGACTCACC	-5'

	TB32047-SC1-9<br>Mutation: Deletion of 1 bp	
5'-	GAA-CGGCACGTCTGAGTGG	-3'
3'-	CTT-GCCGTGCAGACTCACC	-5'

	TB32047-WT or NC<br>NC_000075.7 24759-24778bp	
5'-	GATATCTGATGTGCTAGAGG	-3'
3'-	CTATAGACTACACGATCTCC	-5'

	TB32047-SC2-9<br>Mutation 1: Insertion of 1bp	
5'-	GATAATCTGATGTGCTAGAGG	-3'
3'-	CTATTAGACTACACGATCTCC	-5'

	TB32047-SC2-10<br>Mutation 2: Deletion of 2bp	
5'-	GATAT--GATGTGCTAGAGG	-3'
3'-	CTATA--CTACACGATCTCC	-5'

TB32047-SC2-9<br>Mutation 3: Deletion of 92bp
TB32047-SC2-10<br>Mutation 4: Deletion of 26bp

	PANC-1-WT or NC	
	NC_000006.12 374-393bp	
5’-	CGCCCTGCGCTACGATGCCA	-3’
3’-	GCGGGACGCGATGCTACGGT	-5’

	PANC-1-SC1-1	
	Mutation: Deletion of 5 bp	
5’-	CGCCCTGCGCTACGATG---	-3’
3’-	GCGGGACGCGATGCTAC---	-5’

	PANC-1-SC1-10	
	Mutation: Insertion of 256 bp	
5’-	CGCCCTGCGCTACGATG-CCA	-3’
3-’	GCGGGACGCGATGCTAC-GGT	-5’

	PANC-1-WT or NC	
	NC_000006.12 21295-21314bp	
5’-	CCCACCACGACGTCCACAC	-3’
3’-	GGGTGGTGCTGCAGGTGTG	-5’

	PANC-1-SC2-4	
	Mutation: Deletion of 11 bp	
5’-	C C C - - - - - - - - - - - - C A C A C	-3’
3’-	G G G - - - - - - - - - - - - G T G T G	-5’
