# Supplementary material for: Impact of CRISPR/Cas9-Mediated CD73 Knockout in Pancreatic Cancer
Source: Cancers (Basel). 2023 Oct 3;15(19):4842. doi: 10.3390/cancers15194842 (PMC10572021; doi:10.3390/cancers15194842)
Supplement: Supplementary file 1 [file cancers-15-04842-s001.zip › Supporting document1/Table S2 List of plasmids.pdf]

**Plasmid**	**Source**
lentiCas9-Blast	RRID:Addgene_52962
lentiGuide-Puro	RRID:Addgene_52963
Mouse Brie kinome pooled library	RRID:Addgene_75316
pMDLg/pRRE	RRID:Addgene_12251
pRSV-REV	RRID:Addgene_12253
pMD2.G	RRID:Addgene_12259
pSpCas9(BB)-2A-Puro (PX459) V2.0	RRID:Addgene_62988
