# Supplementary figures and images for: Impact of CRISPR/Cas9-Mediated CD73 Knockout in Pancreatic Cancer
Source: Cancers (Basel). 2023 Oct 3;15(19):4842. doi: 10.3390/cancers15194842 (PMC10572021; doi:10.3390/cancers15194842)

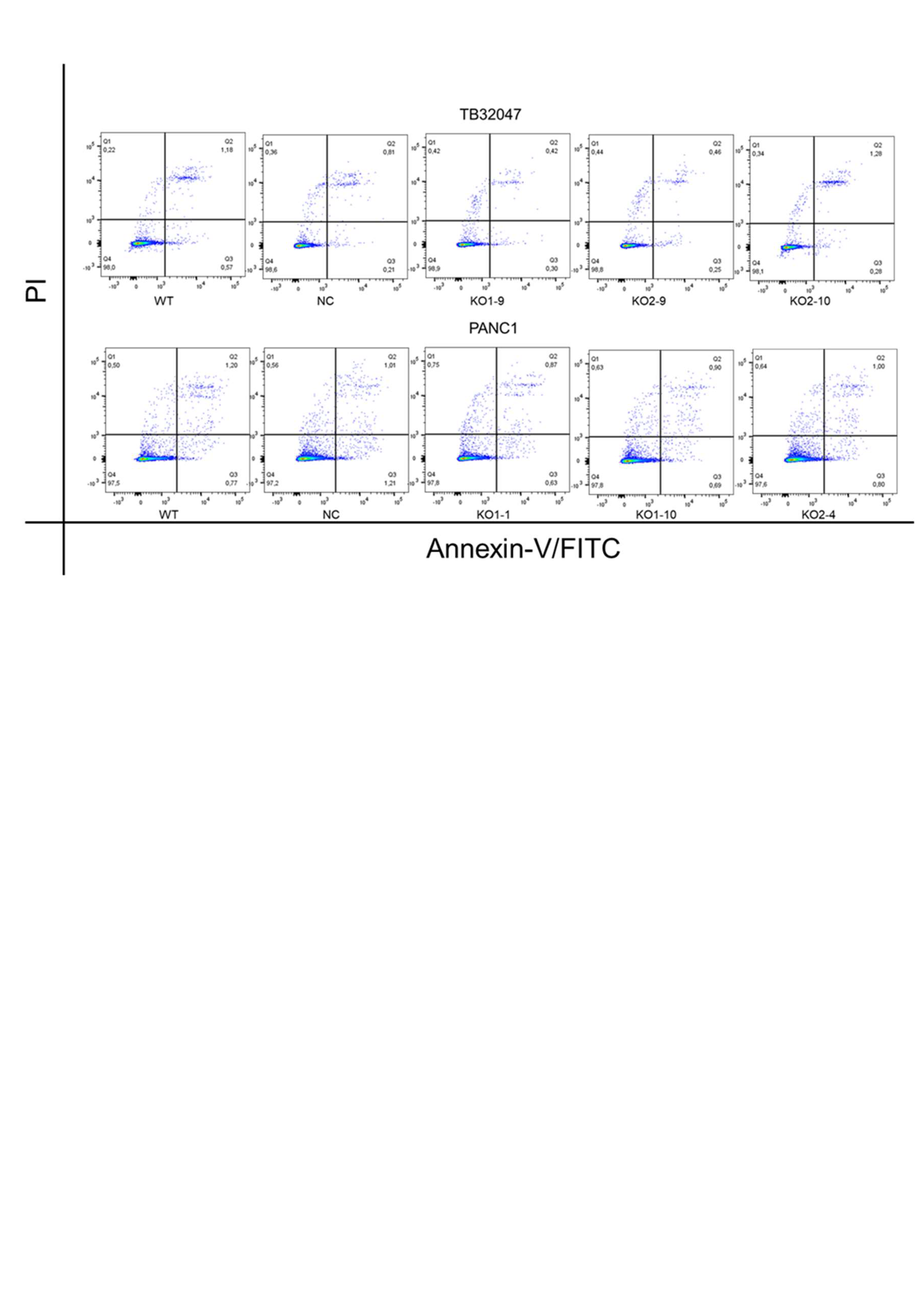

Supplement: Supplementary file 1 [file cancers-15-04842-s001.zip › Supporting document1/Figure S1 Knockout of CD73 did not affect cell apoptosis.tiff]

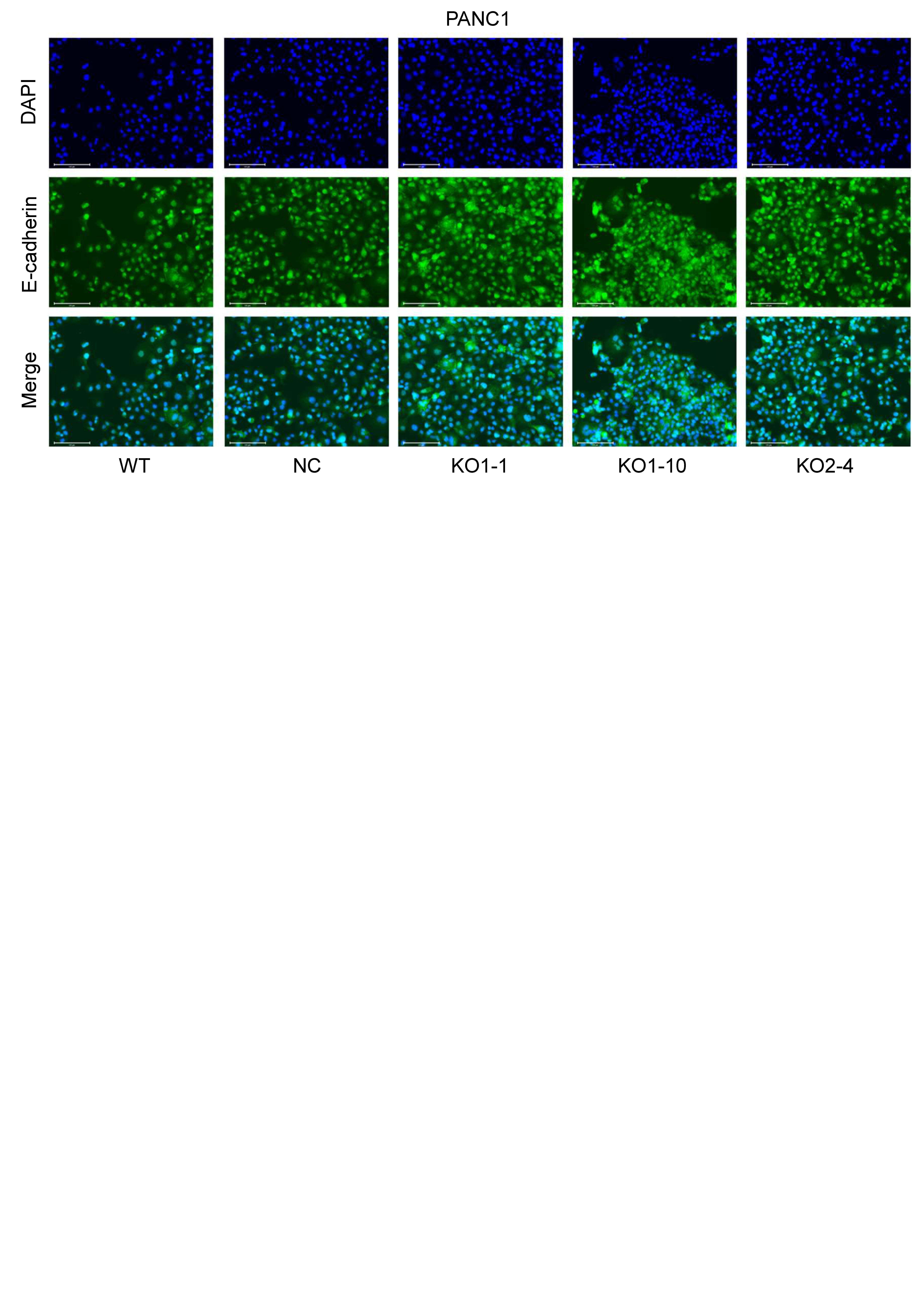

Supplement: Supplementary file 1 [file cancers-15-04842-s001.zip › Supporting document1/Figure S2 E-cadherin did not change in PANC1 cells after CD73 knockout.tiff]
